# Antioxidant and Cytotoxic Activity of New Polyphenolic Derivatives of Quinazolin-4(3H)-one: Synthesis and In Vitro Activities Evaluation

**DOI:** 10.3390/pharmaceutics15010136

**Published:** 2022-12-30

**Authors:** Raluca Pele, Gabriel Marc, Ioana Ionuț, Cristina Nastasă, Ionel Fizeșan, Adrian Pîrnău, Laurian Vlase, Mariana Palage, Smaranda Oniga, Ovidiu Oniga

**Affiliations:** 1 Department of Pharmaceutical Chemistry, Faculty of Pharmacy, “Iuliu Hațieganu” University of Medicine and Pharmacy, 41 Victor Babeș Street, 400012 Cluj-Napoca, Romania; 2 Department of Toxicology, Faculty of Pharmacy, “Iuliu Hațieganu” University of Medicine and Pharmacy, 8 Victor Babeș, 400012 Cluj-Napoca, Romania; 3 National Institute for Research and Development of Isotopic and Molecular Technologies, 400293 Cluj-Napoca, Romania; 4 Department of Pharmaceutical Technology and Biopharmaceutics, Faculty of Pharmacy, “Iuliu Hațieganu” University of Medicine and Pharmacy, 41 Victor Babeș Street, 400012 Cluj-Napoca, Romania; 5 Department of Therapeutical Chemistry, Faculty of Pharmacy, “Iuliu Hațieganu” University of Medicine and Pharmacy, 12 Ion Creangă, 400010 Cluj-Napoca, Romania

**Keywords:** quinazolin-4(3H)-one, phenolic derivatives, hybrid molecules, in vitro evaluation, antioxidant, cytotoxic

## Abstract

The development of hybrid molecules with significant human therapeutic properties is one of the main approaches of pharmaceutical research. One of the most important pharmacophores is the quinazolin-4(3H)-one heterocycle moiety, due to its wide range of biological activities. By its derivatization with polyphenolic compounds, in our previous research, it proved to possess a good antiradical activity of *ortho*-diphenolic derivatives of quinazolin-4(3H)-one. In this study, we developed two new series of compounds, with an additional phenolic group or with a methyl group on the thioacetohydrazone fragment. The methods used to evaluate the activity of the compounds were radical scavenging, reduction of oxidizing reagents and transition metals’ ions chelation assays. Quantum descriptors were also calculated in order to evaluate the influence of substituents and their position on the activity of the compounds. The cytotoxic activity was evaluated using normal human foreskin fibroblast cells (BJ) and two cancerous cell lines, lung adenocarcinoma cells (A549) and prostate carcinoma cells (LNCaP). The results obtained for the pyrogallol derivatives showed a high antioxidant activity compared to ascorbic acid and Trolox. All the synthesized compounds displayed a higher cytotoxicity against the cancerous cell types and a high cytocompatibility with the normal cells. The antioxidant activity was deeply influenced by the addition of the third phenolic group in the synthesized molecules.

## 1. Introduction

The chemical and biological versatility of the quinazolin-4(3H)-one heterocycle moiety is the scientific foundation of our previous research. The following therapeutic characteristics, possessed by these derivatives, are known in the scientific literature: anti-HIV, anticancer, antifungal, antibacterial, antimutagenic, anti-inflammatory, anticonvulsant, CNS depressant, antimalarial, antioxidant, anticoccidial and antileishmanial activity [1,2,3,4,5,6,7,8,9,10].

Today, the medical and pharmaceutical fields are subject to huge progress, with the help of increasingly advanced technology. An interesting observation emphasizes oxidative stress and its effects on the human body. Continuous oxidative stress can be harmful to human cells and can ultimately lead to chronic inflammation. By activating transcription factors, including NF-κB, AP-1, p53, HIF-1α, PPAR-γ, the β-catenin/Wnt pathway, and Nrf2, oxidative stress can lead to the expression of more than 500 different genes. Inflammatory cytokines, chemokines, growth factors, cell cycle regulatory molecules and anti-inflammatory molecules are included as well. Chronic activation of inflammatory pathways can mediate chronic diseases, such as cardiovascular, neurological and pulmonary diseases, diabetes or cancer [1,11,12].

Cancer presents three different stages: initiation, promotion, and progression, with oxidative stress affecting any of these stages. In the first stage, initiation, reactive oxygen species (ROS) may introduce gene mutation and may cause structural alterations in the DNA. In the second stage, promotion, there is an increase in tumor cell proliferation or a decrease in apoptosis. ROS can lead to abnormal gene expression, cell-to-cell communication blockage and second-messenger systems modification. In the last stage, progression, further DNA alterations to the initiated cell population can be added through the mutagenic action of free radicals. Healthy cells, thereby subjected to chronic oxidative stress, can be transformed into cancer cells, through the mechanism presented above. Tumor cell survival, proliferation, chemoresistance, radioresistance, invasion, angiogenesis and stem cell survival are caused in the same way [11,13,14,15].

The antiradical activity of the quinazolin-4(3H)-one is enhanced by its linkage with polyphenolic derivatives, through a thioacetohydrazone fragment, as we described in our previous article on this subject, where 12 compounds, grouped in four series, each with two phenolic groups, were analyzed. The *ortho*-diphenolic compounds had a better antiradical action [16]. The antioxidant activity of the phenolic compounds is already well known in the field, but it can be influenced by the substituents, solvent or matrix [17,18,19,20].

**Scheme 1:**
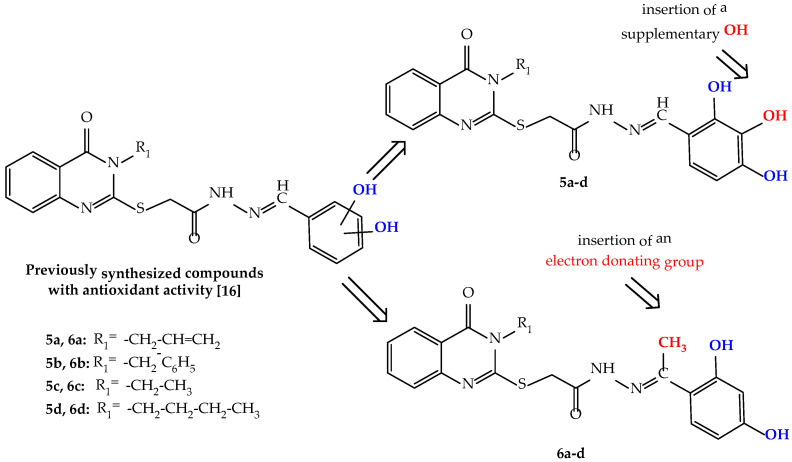
Development of newly polyphenolic derivatives of quinazolin-4(3H)-one.

The purpose of the present research was mainly to investigate the influence exerted on the antioxidant activity of the target molecules by two types of structural derivatizations of previously reported compounds. One development hypothesis evaluated in the present paper is the supplementary insertion of a phenolic OH group (compounds **5a**–**d**). This supplementary OH group could enhance the activity of the compounds by stabilization through intramolecular hydrogen bonding of the resulting radical from the polyphenolic compound, after hydrogen atom abstraction when it manifests its antioxidant activity. The second development hypothesis is represented by the insertion of an electron-donating group (EDG) on the arylidene carbon linked to the polyphenolic moiety (compounds **6a**–**d**). Additionally, we wanted to identify the polyphenolic derivative of quinazolin-4(3H)-one with the lowest toxicity on healthy cells and increased cytotoxicity against two cancerous cell lines (A549 and LNCaP) (Scheme 1).

In the in vitro evaluation of the antioxidant capacity, several antiradical, electron transfer, ferrous and cupric ions chelation assays were used. Cytotoxicity was evaluated using normal human foreskin fibroblasts (BJ) in order to establish the safety of the compounds and two cancerous cell types, namely A549 (lung adenocarcinoma) and LNCaP (prostate carcinoma), to establish a possible anticancerous activity. The theoretical quantum and thermodynamical calculations were also studied for these newly synthesized compounds.

Therefore, we present the chemical synthesis, design, quantum studies and in vitro antioxidant and cell toxicity activities of the new quinazolin-4-one polyphenolic derivatives.

## 2. Materials and Methods

### 2.1. Chemistry

For all synthesis, purification, structural analysis, and in vitro biological activity evaluation, the reagents were purchased from local suppliers and used in accordance with the instructions.

Melting points were measured using a melting point device MPM-H1 (Schorpp Gerätetechnik, Überlingen, Germany), based on the glass capillary method.

The IR spectra were recorded in KBr pellets, under vacuum, with a FT/IR 6100 spectrometer (Jasco, Cremella, Italy).

An Agilent Ion Trap SL mass spectrometer (70 eV) instrument (Agilent Technologies, Santa Clara, CA, USA) was used in negative ionization mode, for the final compounds **5a**–**d** and **6a**–**d**.

^1^H-NMR and ^13^C-NMR spectra were recorded using an Avance NMR spectrometer (Bruker, Karlsruhe, Germany), in dimethylsulfoxide-*d*_6_ (DMSO-*d*_6_). The calibration of the spectrometer was made using tetramethylsilane. The following abbreviations for peak patterns were used to identify the multiplicity of the signals in the ^1^H-NMR spectra: br-broad, s-singlet, d-doublet, dd-double doublet, t-triplet, td-triplet of doublets, q-quartet, quint-quintet, sext-sextet and m-multiplet, respectively. For the signals given by the hydrogen or carbon atoms, to describe the location of the atom in a specific region of the molecule, some abbreviations were used: Q-quinazolin-4(3H)-one, Bz-benzyl and Ar-phenolic benzene ring.

#### 2.1.1. Synthesis of Intermediate Compounds **1a**–**d**, **2a**–**d**, **3a**–**d**

The protocol followed for the synthesis of the intermediate compounds **1a**–**d, 2a**–**d, 3a**–**d** was previously reported by our group. Characterization of the intermediate compounds synthesized in order to obtain the final compounds **5a**–**d** and **6a**–**d** that were previously reported in the literature was consistent with the original papers [21,22,23,24]. The intermediate hydrazides **3a**–**d** used in the present research were obtained during our previous research and were used in the current study without being resynthesized [16].

#### 2.1.2. Synthesis of Compounds **5a**–**d**

Two mmol of the appropriate quinazoline-4-one acetohydrazide **3a**–**d** were suspended in 8 mL of ethanol 96%, in a glass flask. Two mmol of 2,3,4-trihydroxybenzaldehyde and a drop of glacial acetic acid were added. The mixture was refluxed gently, for 3 h, under a condenser. The resulting precipitate was filtered under a vacuum, dried and recrystallized from dioxane.

*2-((3-Allyl-4-oxo-3,4-dihydroquinazolin-2-yl)thio)-N’-(2,3,4-trihydroxybenzylidene)acetohydrazide* (**5a**): white solid; mp = 213 °C; yield = 67.25%; FT IR (KBr) ν_max_cm^−1^: 3475.58 (N–H hydrazone), 3445.69, 3199.81, 3149.19 (phenolic OH), 1696.57, 1646.91 (str C=O), 1558.68 (C=N); MS: m/z = 425.2 (M-1), 441.2 (M + 15); ^1^H-NMR (DMSO-*d*_6_, 500 MHz) δ: 8.326 (s, 1H, =CH-Ar), 8.066 (m, 1H, Q), 7.758 (m, 1H, Q), 7.511 (t, 1H, Q, *J* = 8.5 Hz), 7.452–7.420 (m, 3H, Q), 6.992 (d, 1H, Ar, *J* = 8.5 Hz), 6.816 (d, 1H, Ar, *J* = 8.5 Hz), 5.950 (m, 1H, -CH=), 5.239 (m, 1H, =CH_2_), 5.172 (m, 1H, =CH_2_), 4.758–4.734 (m, 2H, -CH_2_-), 4.129 (d, 2H, -CH_2_-, *J* = 2.25 Hz); ^13^C-NMR (DMSO-*d*_6_, 125 MHz) δ: 167.709 (C=O), 163.012 (C=O), 160.248 (C=N), 156.111 (Ar-OH), 149.077 (Ar-OH), 148.384 (Q), 146.676 (N=CH-Ar), 134.784 (Q), 132.684 (Ar-OH), 131.320 (-CH=), 126.511 (Q), 126.049 (Q), 125.867 (Q), 121.066 (Ar), 118.714 (=CH_2_), 117.699 (Q), 110.728 (Ar), 107.683 (Ar), 45.935 (-CH_2_-), 34.736 (-CH_2_-).

*2-((3-Benzyl-4-oxo-3,4-dihydroquinazolin-2-yl)thio)-N’-(2,3,4-trihydroxybenzylidene)acetohydrazide* (**5b**): yellow solid; mp = 228 °C; yield = 67.32%; FT IR (KBr) ν_max_cm^−1^: 3447.13 (N–H hydrazone), 3222.47, 3046.98, 2918.25 (phenolic OH), 1671.02, 1637.27 (str C=O), 1549.04 (C=N); MS: m/z = 475.4 (M-1), 491.2 (M + 15); ^1^H-NMR (DMSO-*d*_6_, 500 MHz) δ: 8.312 (s, 1H, =CH-Ar), 8.127–8.102 (m, 1H, Q), 7.828–7.765 (m, 1H, Q), 7.490–7.451 (m, 2H, Q), 7.375–7.267 (m, 5H, Bz), 6.812 (d, 1H, Ar, *J* = 8.5 Hz), 6.396 (d, 1H, Ar, *J* = 8.5 Hz), 5.369 (m, 2H, -CH_2_-), 4.108 (s, 2H, -CH_2_-); ^13^C-NMR (DMSO-*d*_6_, 125 MHz) δ: 167.639 (C=O), 162.900 (C=O), 160.814 (C=N), 156.363 (Ar-OH), 148.769 (Ar-OH), 147.341 (Q), 146.704 (N=CH-Ar), 135.547 (Q), 134.945 (Bz), 132.740 (Ar-OH), 128.604 (Bz), 127.484 (Bz), 126.861 (Bz), 126.812 (Q), 126.175 (Q), 125.916 (Q), 121.017 (Ar), 118.714 (Q), 110.700 (Ar), 107.655 (Ar), 46.992 (-CH_2_-), 34.862 (-CH_2_-).

*2-((3-Ethyl-4-oxo-3,4-dihydroquinazolin-2-yl)thio)-N’-(2,3,4-trihydroxybenzylidene)acetohydrazide* (**5c**): yellow solid; mp = 208 °C; yield = 64.23%; FT IR (KBr) ν_max_cm^−1^: 3414.83 (N–H hydrazone), 3186.79, 2970.32, 2931.75 (phenolic OH), 1684.03, 1661.86 (str C=O), 1550.97 (C=N); MS: m/z = 413.7 (M-1), 429.3 (M + 15); ^1^H-NMR (DMSO-*d*_6_, 500 MHz) δ: 8.326 (s, 1H, =CH-Ar), 8.063 (dd, 1H, Q, *J* = 8 and 1 Hz), 7.775–7.710 (m, 1H, Q), 7.441–7.396 (m, 2H, Q), 6.812 (d, 1H, Ar, *J* = 8.5 Hz), 6.396 (d, 1H, Ar, *J* = 8.5 Hz), 4.162–4.104 (m, 4H, -CH_2_- and -CH_2_-), 1.313 (t, 3H, -CH_3_, *J* = 7.25 Hz); ^13^C-NMR (DMSO-*d*_6_, 125 MHz) δ: 167.716 (C=O), 163.047 (C=O), 160.206 (C=N), 155.691 (Ar-OH), 149.049 (C=N), 147.362 (Ar-OH), 146.662 (N=CH-Ar), 134.637 (Q), 132.761 (Ar-OH), 126.378 (Q), 125.951 (Q), 125.797 (Q), 121.099 (Ar), 118.819 (Q), 110.721 (Ar), 107.669 (Ar), 39.342 (-CH_2_-), 34.590 (-CH_2_-), 13.025 (-CH_3_-).

*2-((3-Butyl-4-oxo-3,4-dihydroquinazolin-2-yl)thio)-N’-(2,3,4-trihydroxybenzylidene)acetohydrazide* (**5d**): yellow solid; mp = 239 °C; yield = 67.54%; FT IR (KBr) ν_max_cm^−1^: 3508.85 (N–H hydrazone), 3411.46, 3209.45, 3048.42 (phenolic OH), 1667.16, 1635.34 (str C=O), 1549.52 (C=N); MS: m/z = 441.6 (M-1), 457.6 (M + 15); ^1^H-NMR (DMSO-*d*_6_, 500 MHz) δ: 8.314 (s, 1H, =CH-Ar), 8.070 (dd, 1H, Q, *J* = 8 and 1 Hz), 7.793–7.724 (m, 1H, Q), 7.508 (d, 1H, Q, *J* = 8 Hz), 7.458–7.402 (m, 2H, Q and Ar), 6.809 (d, 1H, Ar, *J* = 8.5 Hz), 4.132–4.066 (m, 4H, -CH_2_- and -CH_2_-), 1.716 (quint, 2H, -CH_2_-), 1.429–1.370 (m, 2H, -CH_2_-), 0.974–0.933 (m, 3H, -CH_3_); ^13^C-NMR (DMSO-*d*_6_, 125 MHz) δ: 167.681 (C=O), 162.984 (C=O), 160.374 (C=N), 155.880 (Ar-OH), 148.979 (Q), 147.320 (Ar-OH), 146.613 (N=CH-Ar), 134.665 (Q), 132.635 (Ar-OH), 126.413 (Q), 125.979 (Q), 125.783 (Q), 120.996 (Ar), 118.749 (Q), 110.679 (Ar), 107.620 (Ar), 43.913 (-CH_2_-), 34.618 (-CH_2_-), 29.585 (-CH_2_-), 19.618 (-CH_2_-), 13.543 (-CH_3_).

#### 2.1.3. Synthesis of Compounds **6a**–**d**

Two mmol of the appropriate quinazoline-4-one acetohydrazide **3a**–**d** were suspended in 8 mL of ethanol 96%, in a glass flask. Two mmol of 2,4-dihydroxyacetophenone and a drop of glacial acetic acid were added. The obtained mixture was refluxed gently, for 4 h, under a condenser. The resulting precipitate was filtered under a vacuum, dried and recrystallized from dioxane.

*2-((3-Allyl-4-oxo-3,4-dihydroquinazolin-2-yl)thio)-N’-(1-(2,4-dihydroxyphenyl)ethylidene)acetohydrazide* (**6a**): pale pink solid; mp = 230 °C; yield = 62.45%; FT IR (KBr) ν_max_cm^−1^: 3475.10 (N–H hydrazone), 3412.90, 3241.27 (phenolic OH), 1697.05, 1660.41, 1607.86 (str C=O), 1550.01 (C=N); MS: m/z = 423.3 (M-1); ^1^H-NMR (DMSO-*d*_6_, 500 MHz) δ: 8.077–8.064 (m, 1H, Q), 7.806–7.745 (m, 1H, Q), 7.519–7.393 (m, 3H, 2H Q and 1H Ar), 6.327 (dd, 1H, Ar, *J* = 9 and 2.5 Hz), 6.242 (d, 1H, Ar, *J* = 2.5 Hz), 5.993–5.917 (m, 1H, -CH_2_-), 5.259–5160 (m, 2H, =CH_2_), 4.748 (d, 2H, -CH_2_-), 4.274 (s, 2H, -CH_2_-), 2.375 (s, 3H, -CH_3_); ^13^C-NMR (DMSO-*d*_6_, 125 MHz) δ: 163.887 (C=O), 163.831 (C=O), 160.542 (C=N), 160.360 (Ar-OH), 160.276 (Ar-OH), 156.328 (N=CH-), 146.627 (Q), 134.651 (Q), 131.313 (-CH=), 129.780 (Ar), 126.448 (Q), 125.958 (Q), 125.671 (Q), 118.784 (=CH_2_), 117.636 (Q), 111.344 (Ar), 106.808 (Ar), 103.119 (Ar), 43.934 (-CH_2_-), 34.352 (-CH_2_-), 13.536 (-CH_3_).

*2-((3-Benzyl-4-oxo-3,4-dihydroquinazolin-2-yl)thio)-N’-(1-(2,4-dihydroxyphenyl)ethylidene)acetohydrazide* (**6b**): yellow solid; mp = 189 °C; yield = 62.54%; FT IR (KBr) ν_max_cm^−1^: 3481.36 (N–H hydrazone), 3412.90, 3272.61 (phenolic OH), 1673.43, 1606.41, 1548.56 (str C=O), 1470.46 (C=N); MS: m/z = 485.2 (M-1); ^1^H-NMR (DMSO-*d*_6_, 500 MHz) δ: 8.135–8.104 (m, 1H, Q), 7.854–7.806 (m, 1H, Q), 7.593 (d, 1H, Q, *J* = 8.25 Hz), 7.508–7.471 (m, 1H), 7.378–7.272 (m, 6H, 5H Bz and 1H Ar), 6.322 (dd, 1H, Ar, *J* = 9 and 2.5 Hz), 6.229 (d, 1H, Ar, *J* = 2.5 Hz), 5.351 (m, 2H, -CH_2_-), 4.240 (s, 2H, -CH_2_-), 2.363 (s, 3H, -CH_3_); ^13^C-NMR (DMSO-*d*_6_, 125 MHz) δ: 166.029 (C=O), 163.747 (C=O), 160.849 (Ar-OH), 160.514 (Ar-OH), 160.269 (C=N), 156.349 (N=CH-), 146.732 (Q), 135.568 (Bz), 134.868 (Q), 129.794 (Ar), 128.597 (Bz), 128.576 (Bz), 127.474 (Bz), 126.826 (Q), 126.749 (Q), 126.119 (Q), 118.658 (Q), 111.323 (Ar), 106.794 (Ar), 103.098 (Ar), 46.859 (-CH_2_-), 34.184 (-CH_2_-), 13.592 (-CH_3_).

*N’-(1-(2,4-dihydroxyphenyl)ethylidene)-2-((3-ethyl-4-oxo-3,4-dihydroquinazolin-2-yl)thio)acetohydrazide* (**6c**): yellow solid; mp = 241 °C; yield = 65.33%; FT IR (KBr) ν_max_cm^−1^: 3469.79 (N–H hydrazone), 3414.35, 3264.89 (phenolic OH), 1697.53, 1655.59, 1606.90 (str C=O), 1549.52 (C=N); MS: m/z = 449.3 (M-1); ^1^H-NMR (DMSO-*d*_6_, 500 MHz) δ: 8.082–8.080 (m, 1H, Q), 7.788–7.742 (m, 1H, Q), 7.499–7.412 (m, 3H, 2H Q and 1H Ar), 6.328 (dd, 1H, Ar, *J* = 9 and 2.5 Hz), 6.248 (d, 1H, Ar, *J* = 2.5 Hz), 4.278 (s, 2H, -CH_2_-), 4.153–4.085 (m, 2H, -CH_2_-), 2.377 (s, 3H, -CH_3_), 1.331–1.277 (m, 3H, -CH_3_); ^13^C-NMR (DMSO-*d*_6_, 125 MHz) δ: 166.211 (C=O), 164.867 (C=O), 160.548 (C=N), 160.283 (Ar-OH), 160.192 (Ar-OH), 156.342 (N=CH-), 146.690 (Q), 134.637 (Q), 129.787 (Ar), 126.287 (Q), 125.937 (Q), 125.671 (Q), 118.840 (Q), 111.351 (Ar), 108.096 (Ar), 102.280 (Ar), 39.342 (-CH_2_-), 34.296 (-CH_2_-), 13.599 (-CH_3_), 12.997(-CH_3_).

*2-((3-butyl-4-oxo-3,4-dihydroquinazolin-2-yl)thio)-N’-(1-(2,4-dihydroxyphenyl)ethylidene)acetohydrazide* (**6d**): yellow solid; mp = 225 °C; yield = 64.79%; FT IR (KBr) ν_max_cm^−1^: 3481.36 (N–H hydrazone), 3420.14, 3238.38 (phenolic OH), 1647.39, 1644.98, 1616.06 (str C=O), 1550.49 (C=N); MS: m/z = 439.3 (M-1); ^1^H-NMR (DMSO-*d*_6_, 500 MHz) δ: 8.081–8.065 (m, 1H, Q), 7.791–7.746 (m, 1H, Q), 7.492 (d, 1H, Q, *J* = 8 Hz), 7.458–7.408 (m, 2H, 1H Q and 1H Ar), 6.381 (dd, 1H, Ar, *J* = 9 and 2.5 Hz), 6.253 (dd, 1H, Ar, *J* = 2.5 Hz), 4.274 (s, 2H, -CH_2_-), 4.102–4.064 (m, 2H, -CH_2_-), 2.375 (s, 1H, -CH_3_), 1.743–1.682 (m, 2H, -CH_2_-), 1.439–1.365 (m, 2H, -CH_2_-), 0.954 (t, 3H, -CH_3_, *J* = 7.25 Hz); ^13^C-NMR (DMSO-*d*_6_, 125 MHz) δ: 164.881 (C=O), 164.188 (C=O), 160.542 (Ar-OH), 160.374 (Ar-OH), 160.283 (C=N), 156.335 (N=CH-), 146.634 (Q), 133.671 (Q), 129.794 (Ar), 126.840 (Q), 126.455 (Q), 125.972 (Q), 118.791 (Q), 111.344 (Ar), 108.103 (Ar), 102.280 (Ar), 43.948 (-CH_2_-), 34.352 (-CH_2_-), 26.330 (-CH_2_-), 19.632 (-CH_2_-), 13.599 (-CH_3_), 13.550 (-CH_3_).

### 2.2. In Vitro Antioxidant, Antiradical and Chelation Assays

The stock solutions (1 mg/mL) of the tested compounds and the controls were obtained, by dissolving the substances in dimethyl sulfoxide (DMSO). Using Jasco V-530, an UV-VIS spectrophotometer (Jasco International Co., Ltd., Tokyo, Japan), we performed the colorimetric determinations in low-volume single-use 10 mm width cuvettes. Solutions of the test compounds did not have absorption maxima near the wavelengths at which these assays were performed.

The assays were performed in triplicate and the results are presented as averages for each assay.

#### 2.2.1. Antiradical Assays

By monitoring the absorbance of reagent in the presence of various amounts of evaluated compounds and controls at λ = 734 nm, a green ABTS**˙**^+^ (2,2′-azinobis-(3-ethylbenzothiazoline-6-sulfonic acid) discoloration assay was performed. The previous protocols reported by our group are based on the initial report of Re et al. [16,25,26]. Using Equation (1), we calculated the ABTS**˙**^+^ scavenging activity of the compounds **5a**–**d** and **6a**–**d**:

(1) $${\mathrm{ABTS}˙}^{+}\;\mathrm{scavenging}\;\left(\%\right)=\frac{\mathrm{control}\;\mathrm{absorbance}-\mathrm{sample}\;\mathrm{absorbance}}{\mathrm{control}\;\mathrm{absorbance}}\times 100$$

The DPPH**˙** radical scavenging assay is based on the transfer of one hydrogen from the analyzed compound to the violet stable free radical DPPH**˙** (2,2-diphenyl-1-picrylhydrazyl), as we convert it into a yellow compound. The absorbance of the reagent mixed with samples presented inversely proportional values to the amount of DPPH**˙** neutralized. The absorbance of the reagent was measured at λ = 517 nm, according to our previous reports [16,26]. The assay was made in the presence of various concentrations of samples. The used protocol is based on the report of Brand-Williams et al. [27]. Using Equation (2), we calculated the DPPH**˙** radical scavenging activity of the compounds **5a**–**d** and **6a**–**d**:

(2) $$\mathrm{DPPH}˙\;\mathrm{scavenging}\;\left(\%\right)=\frac{\mathrm{control}\;\mathrm{absorbance}-\mathrm{sample}\;\mathrm{absorbance}}{\mathrm{control}\;\mathrm{absorbance}}\times 100$$

The scavenging of the NO˙ radical was performed by applying previously reported protocols [16,26]. The in vitro protocol describes the decomposition of sodium nitroprusside in phosphate buffered saline (PBS) at pH = 7.4. The NO˙ radicals would be involved in a Griess reaction if they were not captured by the tested compounds. This reaction results from the formation of an azo dye, with maximum absorption at 546 nm. This azo dye is formed from sulfanilic acid and naphthyl ethylenediamine dichloride [28,29,30]. Using Equation (3), we calculated the NO˙ radical scavenging activity of the compounds **5a**–**d** and **6a**–**d**:

(3) $$\mathrm{NO}˙\;\mathrm{scavenging}\;\left(\%\right)=\frac{\mathrm{control}\;\mathrm{absorbance}-\mathrm{sample}\;\mathrm{absorbance}}{\mathrm{control}\;\mathrm{absorbance}}\times 100$$

#### 2.2.2. Electron Transfer Assays

In the ferric reducing antioxidant potential (FRAP) assay, in comparison with a blank sample prepared from DMSO mixed with FRAP reagent, the absorbance of the solutions was measured at λ = 593 nm. The compounds were tested according to a modified method proposed initially by Benzie and Strain [31]. Using Equation (4), we calculated the reducing antioxidant potential of **5a**–**d** and **6a**–**d** as a percentage of the activity of the reference compounds:

(4) $$\%\;\mathrm{of}\;\mathrm{control}\;\mathrm{activity}=\frac{\mathrm{sample}\;\mathrm{absorbance}}{\mathrm{reference}\;\mathrm{absorbance}}\times 100$$

The reduction capacity of the ferric ion displayed by the tested compounds was determined with the use of a supplementary method. Herein, the iron (III) is not free in the solution as it was in the FRAP assay and is complexed by cyanide ions. The evaluation of the reducing power (RP) was performed at a pH closer to neutral (pH = 6.6); this is different from the FRAP test, which is performed in an acidic environment. Against a blank sample, the absorbances were measured at λ = 700 nm. The followed protocol was based on the adaptation of previously reported protocols [30] and was previously reported by our group [16,32,33]. Using Equation (4), we calculated the reducing power of the **5a**–**d** and **6a**–**d** compounds as a percentage of the reference compounds activity.

Using the phosphomolybdate assay, we determined the total antioxidant capacity (TAC) of the tested compounds. We measured the solutions’ absorbances against a blank sample at λ = 695 nm, using a previously reported procedure by our group [16,32,33], based on initial literature reports [30,34]. Using Equation (4), we calculated the TAC of **5a**–**d** and **6a**–**d** compounds.

The ability to donate electrons was evaluated for the compounds **5a**–**d** and **6a**–**d**, by the CUPRAC (CUPric reducing antioxidant capacity) method. The absorbance was measured against a blank sample, at λ = 450 nm. The assay was performed based on our group’s previous report [16] by the adaptation of the initial report of Alam et al., Özyürek et al. and Apak et al. [30,35,36]. With the use of Equation (4), the cupric ion reduction by compounds **5a**–**d** and **6a**–**d** was expressed as a percentage of the reference compound activity.

#### 2.2.3. Transition Metals’ Ions Chelation Assays

In the protocol used for evaluating the Fe^2+^ chelating ability of the compounds, the absorbances of the solutions were determined at λ = 562 nm, against a blank sample. The method was adapted from the initial report of Benzie and Strain et al. [31], which was previously reported by our group [16,37]. Using Equation (5), we calculated the iron chelation capacities of the **5a**–**d** and **6a**–**d** compounds:

(5) $$\mathrm{iron}\;\mathrm{chelation}\;\left(\%\right)=\frac{\mathrm{control}\;\mathrm{absorbance}-\mathrm{sample}\;\mathrm{absorbance}}{\mathrm{control}\;\mathrm{absorbance}}\times 100$$

With murexide as a chromophoric chelator, we performed the protocol followed for the evaluation of Cu^2+^ chelating activities of the compounds using an adaptation of the method presented by Cesari et al. and Wu et al. [38,39]. Using Equation (6), we calculated the copper chelation capacities of the **5a**–**d** and **6a**–**d** compounds:

(6) $$\mathrm{copper}\;\mathrm{chelation}\;\left(\%\right)=\frac{{\left(\frac{A_{485}}{A_{520}}\right)}_{\mathrm{control}}-{\left(\frac{A_{485}}{A_{520}}\right)}_{\mathrm{sample}}}{{\left(\frac{A_{485}}{A_{520}}\right)}_{\mathrm{control}}}\times 100$$

### 2.3. Theoretical Quantum and Thermodynamical Calculations

The importance of in silico studies has increased over the past few years in the medicinal chemistry research field. By using modern technology, with the help of computers, we can calculate some molecular descriptors, to obtain information of particular significance regarding the explanation or prediction of some experimental results. Through this method, the characteristics of the synthesized compounds (**5a**–**d** and **6a**–**d**) can be determined.

The main molecular descriptors used in the literature are the energy levels of frontier orbitals such as HOMO (highest occupied molecular orbital) and LUMO (lowest unoccupied molecular orbital). They are joined by some thermodynamic calculations, which evaluate the ease of releasing hydrogen atoms from a molecule that acts as an antioxidant compound. When a compound releases hydrogen, the resulting radical must stabilize by internal conjugation, expressed by a lower energy state. This can be calculated as the BDE (bond dissociation enthalpy) of the hydrogen-releasing groups.

The antioxidant activity can be manifested in the case of these new chemically synthesized compounds through the polyphenolic structure, because they present a multitude of phenolic groups.

All theoretical in silico assays reported in this paper were performed following a previously reported protocol [33,40,41]. Using Chimera 1.10.2 (University of California, San Francisco, CA, USA), we generated the depictions of the lowest energy conformation of the compounds [42].

### 2.4. In Vitro Cytotoxicity Activity

#### 2.4.1. Cell Cultures

Normal human foreskin (BJ) fibroblasts and two types of cancer cells, namely A549 (lung adenocarcinoma) and LNCaP (prostate carcinoma) purchased from American Type Culture Collection (Manassas, VA, USA), were used in the experiments. Each cell line was maintained in a different culture medium, depending on the metabolic necessities of the cells. BJ cells were maintained in Dulbecco’s modified Eagle’s medium (DMEM) with low glucose. A549 cells were maintained in DMEM with high glucose (1 g/L), while LNCaP cells were grown in Roswell Park Memorial Institute (RPMI) 1640 medium with high glucose (5 g/L). All media used were supplemented with 10% fetal bovine serum (FBS) and were refreshed every two days. Cells were used in experiments or subcultured when they reached 70–80% confluence.

#### 2.4.2. Experimental

In 100 µL in 96-well plates, a number of 30,000 LNCaP cells, 15,000 A549 cells, and 7500 normal (BJ) cells were seeded. Because the cells have different sizes and volumes, the purpose of the different seedings was to achieve a confluency of 70–80% after 24 h. All types of cells were further exposed to the synthesized compounds (**5a**–**d** and **6a**–**d**) for 24 h, at a concentration ranging from 12.5 to 100 µg/mL. Post-exposure, the media were removed, and the cells were washed with phosphate buffer saline (PBS). Their viability was evaluated using Alamar blue (AB) assay as previously described [43]. Briefly, the cells were incubated with a 200 µM resazurin solution for 4 h. Using a Synergy 2 multi-mode microplate reader, we measured the fluorescence. The measurement was performed at λ_excitation_ = 530/25 nm; λ_emission_ = 590/35 nm. We conducted the experiments using three biological replicates, each one including 6 technical replicates. As negative controls (NC), cells exposed to culture medium containing 0.2% DMSO were used. Compared to the NC (100%), the results were expressed as relative values. We used IC_50_ values for the potency evaluation, and calculated them for each cell line, using the dose-effect curves obtained by fitting the experimental data with a 4-parameter logistic curve.

Mean values ± standard deviation (SD) values of at least three biological replicates were used to calculate experimental data. One-way analysis of variance (ANOVA) with a post hoc Holm–Sidak test was used to statistically examine the data. Using SigmaPlot 11.0 software (Systat, Software Inc., Chicago, IL, USA), we performed the graphical display and data analysis. From a statistical point of view, the results were considered different, at values of *p* < 0.05.

### 2.5. Molecular Properties with Influence on the Pharmacokinetics of Compounds

In order to obtain preliminary information regarding molecular properties which could influence the pharmacokinetics of the compounds, we obtained them using SwissADME [44]. The evaluated molecular properties were the topological polar surface area (TPSA) [45], octanol–water partition coefficient expressed as Moriguchi’s LogP [46], water solubility [47] and violations of the Lipinski’s rule of five [46].

## 3. Results

### 3.1. Chemical Synthesis

**Scheme 2:**
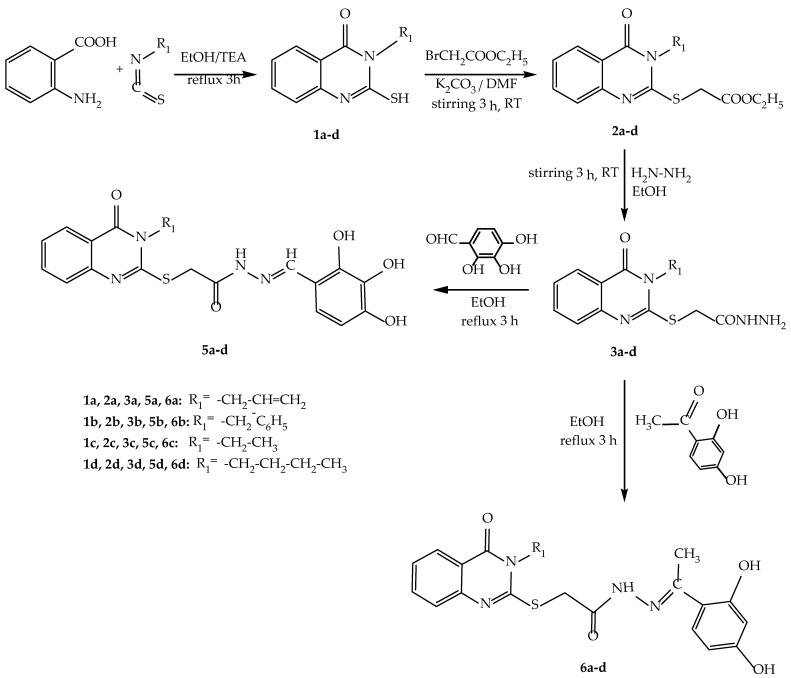
Chemical synthesis of the **5a**–**d** and **6a**–**d** compounds.

A total of eight new final compounds **5a**–**d** and **6a**–**d**, grouped in two series, were synthesized by the condensation of 4-quinazolinon-2-mercapto-acetohydrazides **3a**–**d** with 2,3,4-trihydroxybenzaldehyde or 2,4-dihydroxy-acetophenone, in good yields (all being over 60%). The 4-quinazolinon-2-mercapto-acetohydrazides **3a**–**d** used were obtained in our previous research [16]. All stages of the chemical synthesis are shown in Scheme 2.

The spectral data resulting from the analysis were consistent with the proposed structures. In the IR spectra for all the synthesized compounds **5a**–**d** and **6a**–**d**, the desired signals were revealed.

For the compounds **5a**–**d**, two strong νC=O stretching signals were found between 1637.27–1696.57 cm^−1^: one from the quinazolin-4(3H)-one heterocycle and another one from the thioacetohydrazone linker. The νC=N stretching, from the quinazolin-4(3H)-one heterocycle, had a specific signal between 1550.97–1558.68 cm^−1^. The νN–H stretching bands, from the hydrazone derivate, were between 3414.83–3508.85 cm^−1^. The νO–H stretching, from the phenolic groups, were as wide bands between 2918.25–3445.69 cm^−1^. All the identified signals proved that the condensation took place successfully.

For the compounds **6a**–**d**, between 1548.56–1697.53 cm^−1^, three strong νC=O stretching signals were found: one from the quinazolin-4(3H)-one heterocycle, one from the thioacetohydrazone linker and another from the 2,4-dihydroxy-acetophenone. Between 1550.49–1570.46 cm^−1^, the νC=N stretching had a specific signal from the quinazolin-4(3H)-one heterocycle. Between 3469.79–3481.36 cm^−1^, the νN–H stretching bands had a signal from the hydrazone derivate. Between 3238.38–3420.14 cm^−1^, the νO–H stretching bands were wide due to the presence of phenolic groups. All the signals were in accordance with the proposed structures.

In the MS spectra of the synthesized compounds **5a**–**d** and **6a**–**d,** the corresponding molecular mass peaks were identified.

In the ^1^H-NMR spectra of the **5a**–**d** and **6a**–**d** compounds, all the expected signals of all protons were identified, with the corresponding multiplicity. In the ^13^C-NMR spectra of compounds, the expected signals corresponding to the carbon atoms were identified. All the signals were in the expected region of each spectrum.

For compounds **5a**–**d** and **6a**–**d**, the graphic depictions of the recorded spectra are provided in the Supplementary Materials (Figures S1–S32).

### 3.2. In Vitro Antioxidant, Antiradical and Chelation Assays

The antioxidant potentials of compounds **5a**–**d** and **6a**–**d** were evaluated based on their direct antiradical activity and reduction of oxidized reagents and, complementarily on their transition metals’ chelation activity. The in vitro protocols applied for the current evaluations were performed at a semi-microscale level, according to our group’s previous reports and using reference compounds [32,33]. All determinations were performed in triplicate and results are presented as averages.

#### 3.2.1. Antiradical Assays

The antiradical potential of compounds **5a**–**d** and **6a**–**d** was evaluated spectrophotometrically as a capacity to scavenge the ABTS˙^+^, DPPH˙ and NO˙ radicals.

##### ABTS˙^+^ Radical Scavenging Assay

Compounds **5a**–**d** and **6a**–**d** were screened for their ABTS˙^+^ radical scavenging capacity and the results obtained are presented in Table 1. Trolox and ascorbic acid were used as positive controls. The most active compounds were **5a**, **5c** and **5d,** having lower IC_50_ values than those of the antioxidant reference drugs.

##### DPPH˙ Radical Scavenging Assay

The DPPH˙ scavenging activity of the compounds **5a**–**d** and **6a**–**d** was evaluated spectrophotometrically and the obtained results are presented in Table 2. The reference antioxidants were ascorbic acid and Trolox. Compounds **5a**, **5c** and **5d** had the best activity.

##### NO˙ Radical Scavenging Assay

The NO˙ antiradical potential of compounds **5a**–**d** and **6a**–**d** and gentisic acid was evaluated spectrophotometrically, based on the Griess reaction; the obtained results are presented in Table 3. Compounds **5a** and **5c** had the best ability to scavenge this radical.

#### 3.2.2. Electron Transfer Assays

Using the FRAP, TAC, RP and CUPRAC spectrophotometric assays, we determined the antioxidant capacity of compounds **5a**–**d** and **6a**–**d** as the capacity of donating electrons.

##### Ferric Reducing Antioxidant Power (FRAP)

Ferrous ions resulting from the reduction of ferric ions in the presence of an antioxidant compound form a blue-colored complex (Fe^2+^-TPTZ) at pH = 3.6 with tripyridyltriazine (2,4,6-tris(2-pyridyl)-s-triazine). The intensity of the blue complex formed is proportional to the number of ferrous ions. The results obtained for the FRAP assay are presented in Table 4. Compounds **5a**, **5c** and **5d** expressed a good electron donation capacity.

##### Phosphomolybdate Assay for Total Antioxidant Capacity (TAC)

At acidic pH, the tested compounds transfer one electron to Mo^6+^ to reduce it to Mo^5+^. The higher amount of Mo^5+^ green phosphate complex that results, the more active the tested compound is. The results of the TAC assay are presented in Table 4. The obtained results of the TAC assay proved the excellent electron-donating capacity of compounds **5a**, **5b** and **5c**.

##### Reducing Power (RP) Assay

The evaluated compounds can reduce ferricyanide to ferrocyanide, in the presence of ferric ions, resulting in the Perl’s Prussian which gives blue. The higher the percentage of reducing power, the higher was the absorbance that was measured due to a higher amount of colored complex resulting from the reaction. The results are presented in Table 4. Compounds **5a**, **5b** and **5c** displayed a good activity.

##### Cupric Reducing Antioxidant Capacity (CUPRAC) Assay

The Cu^2+^ ions are reduced to Cu^+^ ions by the electron-donating compounds. The Cu^+^ ions are chelated by neocuproine, giving a colored complex with absorbance proportional to the quantity of Cu^+^ ions that resulted. The results of the CUPRAC assay are presented in Table 4. Compounds **5a**, **5c** and **5d** displayed excellent activity.

#### 3.2.3. Transition Metals’ Ions Chelation Assays

##### Fe^2+^ Chelation Assay

The chelating capacity of the tested compounds of Fe^2+^ was evaluated based on their competition for Fe^2+^ with ferrozine. A decrease in the resulting absorbance indicated that the ferrous ions were sequestered by the evaluated compounds. The result of the Fe^2+^ chelation assay is presented in Table 5. The most active compounds were **5a**, **5c** and **5d**.

##### Cu^2+^ Chelation Assay

The evaluation of the Cu^2+^ chelation activity of compounds was made based on the competition for Cu^2+^ with murexide. The results are presented in Table 6. The chelating activity of compounds for the cupric ions found in the current assay is significant for the pyrogallol derivatives, being quite close to EDTA-Na_2_.

### 3.3. Theoretical Quantum and Thermodynamical Calculations

**Figure 1:**
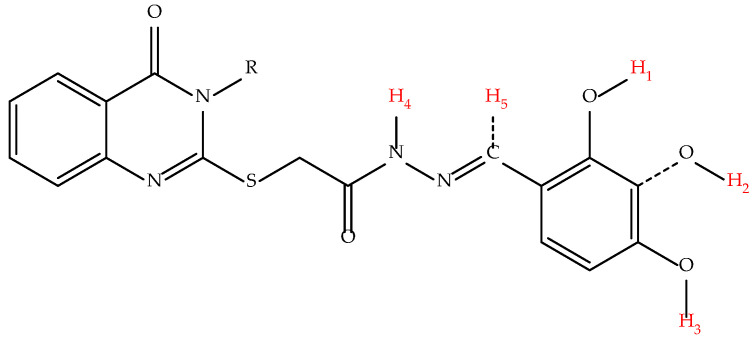
General structure representing the possible sites of compounds **5a**–**d** and **6a**–**d** to release hydrogen atoms (numbered H_1_–H_5_) and to neutralize radicals.

The highest occupied molecular orbital (HOMO) is the parameter that indicates a good capacity for electron donation of the molecule which is due to the susceptibility of a molecule to be attacked by electrophilic species. The energy of the lowest unoccupied molecular orbital (LUMO) is the parameter that is related to its susceptibility to be attacked by nucleophilic species and to its electron affinity. The general structure representing the possible sites of **5a**–**d** and **6a**–**d** molecules to release hydrogen atoms is shown in Figure 1.

The energy levels of HOMO and LUMO are presented in Table 7, together with the bond dissociation energies (BDEs). The BDEs of the sites in the molecules can yield hydrogen atoms (numbered H_1_–H_5_) and are derived from the in-silico calculations. The depiction of HOMO and LUMO as well as the depiction of the spin density maps of the phenol group radicalization are presented in Supplementary Materials (Tables S1 and S2).

Regarding HOMO energy levels, the identified values were lower for the pyrogallol-derived molecules, compounds **5a**, **5b**, **5c** and **5d,** (between −5.53 eV and −5.51 eV) and slightly higher for compounds **6a**, **6b**, **6c** and **6d** (between −5.42 eV and −5.39 eV). In the case of the **5b** and **5c** molecules, the lowest HOMO orbital was identified, with an energy level of −5.53 eV. The highest HOMO orbital was identified in the case of the **6b** and **6d** molecules at −5.39 eV.

The lowest values for the LUMO energy levels were identified in **5b** and **6b** (−1.64 eV). Both compounds were substituted at the N_3_ atom from the quinazolin-4(3H)-one ring with a benzyl moiety. The LUMO energy level in the **5b** and **6b** compounds was significantly influenced by this substituent, because in all the other compounds the values were quite similar (between −1.60 eV and −1.55 eV), energies being a little smaller in the compounds **5a**, **5b**, **5c** and **5d** compared to the compounds **6a**, **6b**, **6c** and **6d**.

The HOMO–LUMO gap was found to be higher in the case of the pyrogallol-derived compounds **5a**, **5b**, **5c** and **5d** (between 3.89 eV and 3.93 eV), while in the case of the 2,4-dihydroxy-acetophenone derivatives **6a**, **6b**, **6c** and **6d,** the energy difference between the two frontier orbitals was found to be between 3.75 eV and 3.84 eV.

Overall, the most susceptible group to release hydrogen atoms is the meta-hydroxy (site H_3_) found in compounds **5a**, **5b**, **5c** and **5d** (pyrogallol-derived compounds), with a BDE ranging from 71.98 kcal/mol to 72.00 kcal/mol.

The next type of groups in ascending order of BDE that could yield hydrogen atoms are *ortho*-hydroxy (site H_1_) in the **5a**, **5b**, **5c** and **5d** compounds, with a BDE ranging from 77.95 kcal/mol to 78.08 kcal/mol. The next type of group that releases hydrogen atoms is the *para*-hydroxy (site H_3_), with similar values in both series of compounds, ranging between 78.26 kcal/mol and 79.49 kcal/mol.

Interestingly, the *ortho*-OH group (site H_1_) in the case of **6a**, **6b**, **6c** and **6d** (dihydroxy-acetophenone derivatives) had a significantly higher BDE compared to those in pyrogallol-derived compounds **5a**, **5b**, **5c** and **5d**, relative to the same site H_1_, values ranging from 84.44 kcal/mol to 84.64 kcal/mol. Most likely, in the case of the dihydroxy-acetophenone derivatives compounds, compared to the pyrogallol-derived compounds, this is a consequence of the insertion of the methyl group on the azomethine carbon atom and the disappearance of the phenolic group from the meta position.

On the next level, in terms of ease of release of hydrogen atoms, is the N-H group (site 4). Hydrogen atoms could be more easily released from this site by the compounds from the dihydroxy-acetophenone derivatives compounds, having a BDE ranging from 83.12 kcal/mol to 83.39 kcal/mol, lower than those from the pyrogallol-derived compounds, which had a significantly higher BDE for the N-H bond, ranging from 86.01 kcal/mol to 89.91 kcal/mol.

Abstraction of hydrogen atoms from the azomethine group (site H_5_) would be difficult, because the BDE of the C-H bond was much higher, with values ranging from 100.98 kcal/mol to 106.64 kcal/mol. The BDE computed for the C-H bonds from the allyl group of compounds **5a** and **6a** was found to be more than 105 kcal/mol, which makes the allyl group inert in terms of hydrogen atom abstraction for neutralization of an external radical.

In all compounds, the *ortho*-hydroxy phenol made a hydrogen bond as a donor to the imine nitrogen atom from the hydrazone as an acceptor. The hydrazide−hydrazone group had a negative effect on the hydrogen release from the *ortho*-OH groups, due to the intramolecular hydrogen bonding, the BDE of OH from H_1_ site being higher than ones from H_2_ or H_3_. On the other hand, compounds from the pyrogallol-derived series were strongly favorized by the internal hydrogen bonding. The pseudo-polycyclic rings that appear due to the internal hydrogen bonding favored the stabilization by the extended internal conjugation of the resulting radicals after hydrogen atom release.

The conformation with the lowest energy of the **5a**–**d** and **6a**–**d** compounds with the intramolecular hydrogen bonds is presented in Table 8.

### 3.4. In Vitro Cytotoxicity Activity

**Figure 2:**
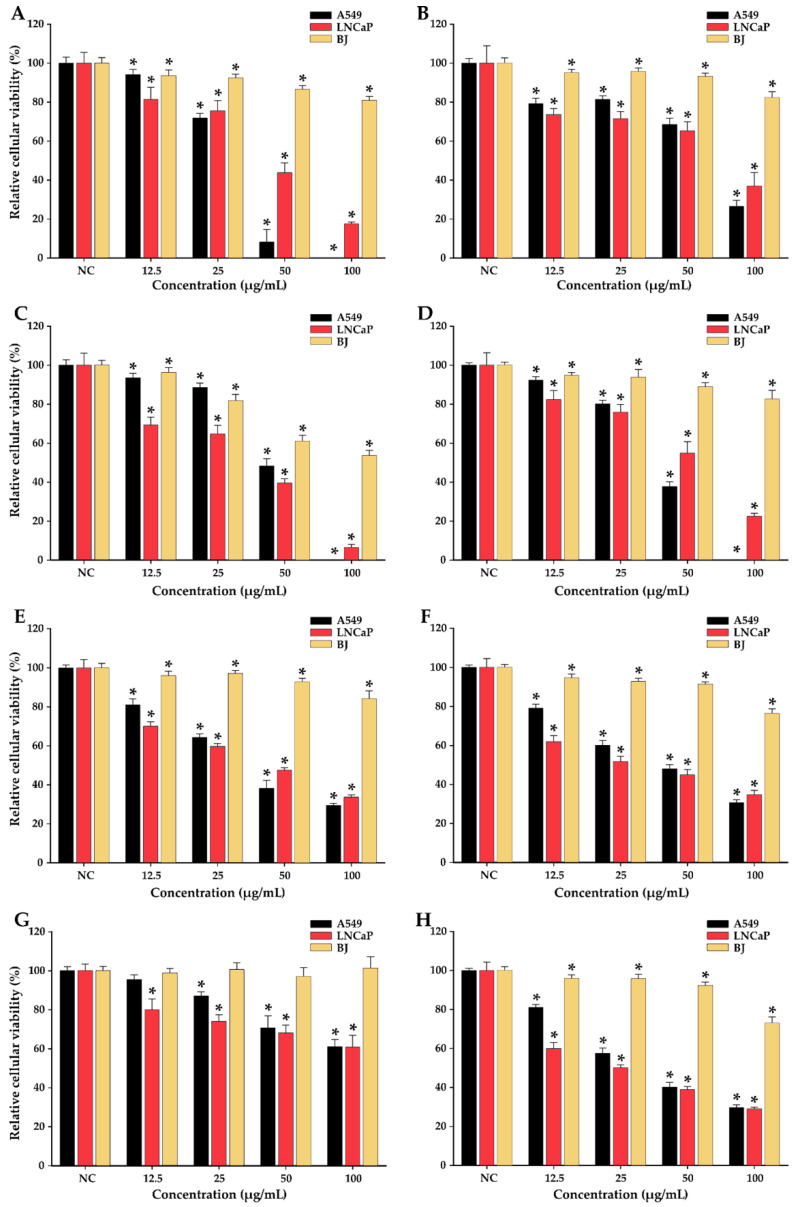
Cytotoxic effect of **5a** (**A**), **5b** (**B**), **5c** (**C**), **5d** (**D**), **6a** (**E**), **6b** (**F**), **6c** (**G**) and **6d** (**H**) after 24 h exposure on A549, LNCaP and BJ cells. The results are presented as relative means ± standard deviations of three biological replicates (six technical replicates for each biological replicate). Viability data were expressed as relative values, where the negative control (NC) is 100%. Asterisks (*) indicate significant differences (*p* < 0.05) compared to NC.

**Figure 3:**
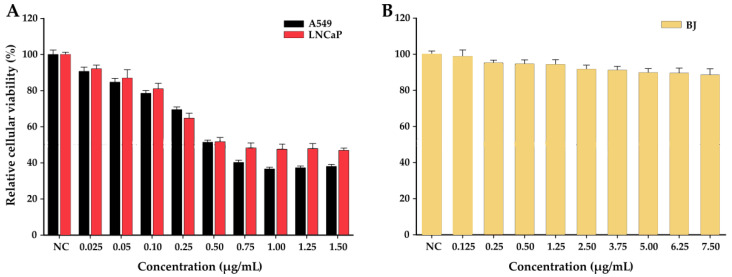
Cytotoxic effect of doxorubicin (**A**,**B**) after a 24 h exposure on A549, LNCaP and BJ cells.

The cytotoxic effects of the synthesized compounds **5a**–**d** and **6a**–**d** were evaluated on two cancerous cell lines (A549, LNCaP), in parallel with normal fibroblast cells (BJ). Independently of the concentration tested, all the compounds presented higher cytotoxicity towards the cancerous cell phenotypes (Figure 2). The citotoxiciy of doxorubicin, used as a standard agent, is presented in Figure 3.

The IC_50_ values were calculated for all the compounds towards doxorubicin (Table 9). The results indicated that at all concentrations tested, the type of cells and the compounds tested had a statistically significant contribution to the recorded viability.

In case of A549, the potency of the compound varied in the order **5a** > **6d** > **6a** > **5d** > **6b** > **5c** > **5b** > **6c**, while in the case of LNCaP the order was **6d** > **6b** > **5c** > **6a** > **5a** > **5d** > **5b** > **6c**. A three-way ANOVA with the cell type (A549, LNCaP, and BJ), exposure dose (12.5, 25, 50, and 100 µg/mL), and type of compounds (**5a**–**d** and **6a**–**d**) as variables and the measured viability as a response was performed to evaluate if there are any statistical differences between the conditions.

Except for compound **6c** which presented the lowest toxicity on all three types of cell lines, all compounds induced statistically significant cytotoxicity starting from the lowest dose of 12.5 µg/mL on all three cellular lines.

Interestingly, different anticancerous potencies were observed for the synthesized compounds between the two cell lines employed. For the A549 cells, compounds **5a**, **5d**, **6a** and **6d** which shared the same substituents (R_1_) (-CH_2_-CH=CH_2_ and -CH_2_-CH_2_-CH_2_-CH_3_) were more cytotoxic, while in the case oF LNCaP cells no such observations could be made because the four most cytotoxic compounds had different substituents. Compounds **5b** and **6c** had a similar toxicity pattern on both cancerous cell types.

All compounds presented higher cytotoxicity on the LNCaP cells than on the A549 cells at an exposure dose of 12.5 µg/mL. This effect could be related to the weak attachment of the LNCaP cells to the plastic substrate, the presence of the substances favoring the detachment of the cells and thus inducing an apparent decrease in the recorded viabilities.

### 3.5. Molecular Properties with Influence on the Pharmacokinetics of Compounds

For the compounds **5a**–**d** and **6a**–**d**, molecular properties were computed in silico and are presented in Table 10. The molecular weight of compounds is found between 412.46 and 476.50, all of them being under 500, respecting the rule proposed by Lipinski [46]. Compounds **5b** and **6b** possessing a bulky benzylidene ring (R_1_) have the highest molecular weight in the present series. Compounds from the **a** series that have an extra phenol group have a molecular weight two units higher than those from the **b** series, which have an extra methyl group.

The number of rotatable bonds in all compounds are under 10, respecting the rule proposed by Lipinski. No differences between the compounds from the two series are identified by the proposed structural modifications.

The number of hydrogen bond acceptors (HBA) and hydrogen bond donors (HBD) is in direct relationship with the structure of the compounds in the two series. Insertion of the supplementary phenol group in the **a** series of compounds compared with the **b** series of compounds leads to their increase by one unit. Regarding the HBA and HBD parameters, all compounds pass the rule proposed by Lipinski.

Insertion of the supplementary phenol group led to the increase in the polar surface in the case of compounds from series **a** (162.34 Å^2^), compared to those from series **b** (142.11 Å^2^). The R_1_ substituent has no influence on the polar surface of the compound, because of its lipophilic properties, without any polar groups. It influences the lipophilicity of the compounds, in the order ethyl < butyl < allyl < benzyl. When we corroborate all the molecular properties of compounds **5a**–**d** and **6a**–**d**, it can be concluded that none of them violates any of Lipinski’s rules.

Solubility in water is strongly influenced by the chemical structure of the compounds in the present study. The supplementary phenol group from the series **a** of compounds increases the solubility of the compounds in water, compared to the **b** series of compounds. For the compounds that possess the same R_1_, the solubility in water of the compounds from series **a** is more than double that of those from series **b**. R_1_ strongly influences the solubility of compounds in water. The most soluble are those in which R_1_ is small (ethyl), and with its increase, the solubility of the compounds decreases, the most insoluble being those where R_1_ is benzyl.

## 4. Discussion

### 4.1. Chemical Synthesis

The synthesis and characterization of the intermediate compounds **1a**–**d**, **2a**–**d** and **3a**–**d** were previously reported by our group [16].

In the final step of the chemical synthesis, compounds **5a**–**d** and **6a**–**d** were synthesized by refluxing in ethanol the previously obtained 4-quinazolinon-2-thioacetohydrazides **3a**–**d** with 2,3,4-trihydroxybenzaldehyde or 2,4-dihydroxy-acetophenone. The spectral analysis performed indicated successful obtention of the desired compounds.

### 4.2. In Vitro Antioxidant, Antiradical and Chelation Assay

The results of the in vitro evaluations performed indicated that the **5a**–**d** series of compounds (pyrogallol derivatives) exhibit a higher antioxidant activity than the **6a**–**d** series of compounds (resorcinol derivatives). It can be concluded that the insertion of the third phenol group (in the **5a**–**d** series of compounds) was much more effective than the insertion of the supplementary methyl group on the azomethine carbon (in the **6a**–**d** series of compounds).

#### 4.2.1. Antiradical Assays

In the antiradical assays (ABTS˙^+^, DPPH˙ and NO˙), the obtained results displayed compounds **5a, 5c** and **5d** as the most active of the two synthesized series of compounds. In comparison with the radical scavenging activity of the reference antioxidants used, the activity of **5a, 5c** and **5d** was higher.

#### 4.2.2. Electron Transfer Assays

The pyrogallol derivatives **5a**, **5b**, **5c** and **5d** were the most active compounds. The results of this assay revealed a better antioxidant potential for these derivatives than that of ascorbic acid and Trolox, used as reference antioxidants.

#### 4.2.3. Transition Metal Ions Chelation Assays

The chelating activity of compounds for the ferrous ions was higher for the **5a, 5c** and **5d** compounds. The Cu^2+^ chelation activity was significant for the **5a**, **5b** and **5d** compounds, being quite close to that of EDTA-Na_2_.

According to the structure–activity relationship, the pyrogallol derivatives had better chelating properties.

### 4.3. Theoretical Quantum and Thermodynamical Energy Calculations

The BDE (bond dissociation enthalpy) of the hydrogen-releasing groups to neutralize external radicals was the second theoretical important descriptor analyzed in this research, because the energy of the frontier orbitals failed to make a correlation with the antioxidant activity of the compounds. The evaluation of the BDE indicated that the most susceptible compounds to release a hydrogen atom are those from the **5a**–**d** series (pyrogallol-derived compounds) from the newly introduced phenol group from meta. This supplementary group influences the antioxidant activity of the compounds not just per se, but it reduces the ease of release of hydrogen atoms from the phenol groups from *ortho* and *para*, from the **5a**–**d** compounds, compared to the **6a**–**d** compounds, that lack the supplementary OH group in meta, but possess a supplementary methyl group on the azomethine. It can be concluded that in terms of antioxidant activity expressed as hydrogen atoms release, the beneficial effect of the insertion of the third phenol group is much higher than the insertion of the supplementary methyl group.

### 4.4. In Vitro Cytotoxicity Activity

As a next step in the biological activity evaluation of the synthesized compounds, their possible antitumoral activity towards two different cancerous cell lines in parallel with normal cells was determined. All compounds (**5a**–**d** and **6a**–**d**) were evaluted in the concentration range of 12.5–100 µg/mL after a 24 h exposure, by the Alamar blue assay as previously described [43]. The normal cell line was included to verify if the synthesized compounds display a selective toxicity towards the cancerous phenotype. The cytotoxic effects of the compounds were compared with those of a reference anticancerous substance, namely doxorubicin.

The compounds exhibited lower cytotoxicity on the normal fibroblast cell line and higher cytotoxicity on the cancerous cell lines. With the exception of compound **5c** that decreased the cellular viability in BJ cells by approximately 40% at the highest concentration tested, all the other compounds displayed high cytocompatibility on normal cells. At the highest concentration tested, the recorded viabilities for BJ ranged from 50 to 100%, while in the case of A549 and LNCaP the viabilities ranged between 0 and 60% and 5 and 60%, respectively.

Different cytotoxicity patterns were observed for the two series of compounds (**5a**–**d** and **6a**–**d**), with compounds **6a**–**d** inducing more pronounced toxicity than the other compounds at lower concentrations. Compounds **5a**–**d** induced higher cytotoxicity at intermediary and high doses.

As the IC_50_ values of doxorubicin are much lower than the IC_50_ values of the **5a**–**d** and **6a**–**d** compounds (approximately 100 times), a potential anticancerous effect of the tested compounds is not promising.

### 4.5. Molecular Properties with Influence on the Pharmacokinetics of Compounds

The increase in the size of the lipophilic substituent at the level of the nitrogen atom of quinazolone (R_1_) leads to an increase in the molecular weight, an increase in lipophilicity and a decrease in the water solubility of the compounds. On the contrary, the smaller the R_1_ lipophilic substituent, the lower the molecular mass, the lower the lipophilicity and the higher the solubility in water. It can be seen that the insertion of the third phenolic OH group in compounds **5a**–**d** leads to an increase in the polarity of the compounds, a decrease in lipophilicity and an increase in their solubility in water, compared to the series of compounds **6a**–**d**.

The way in which the substitution influences the properties influencing the pharmacokinetics of the compounds in the present study follows a normal trend for medicinal chemistry and the design of new bioactive compounds. The variation in the molecules of R_1_ and arylidene can bring a significant variation in the properties of compounds. For example, the compound **5c** is 24 times more soluble in water than **6b**. On the other hand, compound **6b** being more lipophylic and less polar than compound **5c** can have a better penetration through cell membranes.

## 5. Conclusions

Through this study of some new polyphenolic derivatives of quinazolin-4(3H)-one, the synthesis, characterization and in-vitro antioxidant and cytotoxic activity evaluation are presented.

Compounds **5a**, **5b**, **5c**, and **5d** were the most active, following the evaluation of the antioxidant activity. Their antiradical potential was better than that of ascorbic acid and Trolox, used as reference antioxidants, for these pyrogallol derivatives.

The HOMO–LUMO gap was higher in the case of the pyrogallol-derived compounds **5a**, **5b**, **5c** and **5d**, while in the 2,4-dihydroxy-acetophenone derivatives **6a**, **6b**, **6c** and **6d**, the energy difference between the two frontier orbitals was lower. The most susceptible group to release hydrogen atoms was meta-hydroxy (site H_3_), which is found in the **5a**, **5b**, **5c** and **5d** compounds (pyrogallol-derived compounds).

The cytotoxic activity was evaluated on three different cell lines (BJ, A549 and LNCaP). The compounds **6a**–**d** induced a higher toxicity than the other compounds on A549 cells and LNCaP cells at lower concentrations, while the compounds **5a**–**d** induced higher cytotoxicity at intermediary and high doses. In comparison with cancerous cells, higher viabilities were observed on the normal cells after exposure to the synthesized compounds. Thus, in a therapeutic hypothesis, the new synthesized compounds are cytocompatible towards normal cells and healthy tissue.

The results of the in-vitro studies performed on these compounds confirm that the antioxidant activity is strongly influenced by the presence of the third phenolic-OH group in the molecular structure. The trihydroxilated hybrid derivatives exhibited lower cytotoxicity on the normal fibroblast cell line and activity on the cancerous cells lines depending of the concentrations of the compounds. The methyl group inserted in the arylidene carbon did not increase the antioxidant potential of the compounds, but influenced the cytotoxicity activity on cell lines.

## Figures and Tables

**Table 1:** Results of the ABTS**˙**^+^ scavenging assay.

Compound	% of ABTS˙^+^ Scavenging							IC_50_ (µg/mL)	IC_50_ (µM)
	0.83 µg/mL	1.67 µg/mL	3.33 µg/mL	4.99 µg/mL	6.66 µg/mL	9.99 µg/mL	13.32 µg/mL		
**5a**	38.14	51.47	72.00	+	+	+	+	1.65	3.87
**5b**	34.65	49.32	68.62	85.98	+	+	+	1.91	4.01
**5c**	39.05	52.25	72.57	+	+	+	+	1.60	3.86
**5d**	41.98	52.25	67.46	81.06	+	+	+	1.55	3.50
**6a**	27.03	30.08	37.19	42.27	50.03	60.55	70.24	7.17	16.89
**6b**	26.26	33.32	44.60	55.88	68.23	90.98	+	4.13	8.70
**6c**	29.08	34.73	43.19	54.47	68.58	91.60	+	4.06	9.84
**6d**	29.08	33.32	40.37	50.24	57.59	75.41	85.79	5.17	11.74
**Ascorbic acid**	60.97	73.24	87.73	+	+	+	+	2.01	11.41
**Trolox**	38.66	53.16	66.54	94.57	+	+	+	4.66	18.62

+ represents more than 95%.

**Table 2:** Results of the DPPH**˙** scavenging assay.

Compound	% of DPPH˙ Scavenging								IC_50_ (µg/mL)	IC_50_ (µM)
	1.25 µg/mL	2.5 µg/mL	3.75 µg/mL	5 µg/mL	7.5 µg/mL	10 µg/mL	12.5 µg/mL	15 µg/mL		
**5a**	41.09	49.94	54.93	62.06	73.89	+	+	+	2.76	6.47
**5b**	37.55	45.21	51.00	56.36	69.66	81.12	+	+	3.62	7.60
**5c**	44.32	55.40	64.87	73.44	90.15	+	+	+	1.85	4.46
**5d**	41.12	53.03	63.53	71.70	87.99	+	+	+	2.18	4.93
**6a**	-	-	-	-	-	-	12.28	21.34	>15	>30
**6b**	-	-	-	-	-	-	-	11.59	>15	>30
**6c**	-	-	-	-	-	-	10.11	14.89	>15	>30
**6d**	-	-	-	-	-	-	10.99	13.87	>15	>30
**Ascorbic acid**	35.11	47.45	55.71	64.21	79.16	94.39	+	+	2.83	16.07
**Trolox**	17.01	28.53	40.42	53.87	75.85	94.85	+	+	4.68	18.70

- represents less than 5%; + represents more than 95%.

**Table 3:** Results of the NO˙ scavenging assay.

Compound	% of NO˙ Scavenged
**5a**	38.39
**5b**	36.75
**5c**	43.21
**5d**	35.14
**6a**	31.82
**6b**	33.31
**6c**	30.62
**6d**	30.71
**Gentisic acid**	48.14

**Table 4:** Results of the Ferric Reducing Antioxidant Potential (FRAP), Total Antioxidant Capacity (TAC), Reducing Power (RP) and Cupric Reducing Power (CUPRAC) assays, expressed as % activity of ascorbic acid and Trolox.

Compound	% of Activity of Ascorbic Acid				% of Activity of Trolox			
	FRAP	TAC	RP	CUPRAC	FRAP	TAC	RP	CUPRAC
**5a**	86.18	91.43	63.20	132.79	98.88	176.42	92.09	126.56
**5b**	67.05	81.34	58.80	97.31	76.93	156.96	85.68	92.75
**5c**	83.40	84.73	49.93	116.41	95.70	163.49	72.75	110.95
**5d**	80.80	61.09	49.54	111.66	92.71	117.87	72.18	106.43
**6a**	22.81	48.91	21.09	13.46	26.17	94.38	30.73	12.83
**6b**	18.51	52.36	19.91	23.74	21.24	101.03	29.01	22.63
**6c**	25.85	57.03	19.89	30.52	29.65	110.05	28.98	29.09
**6d**	24.36	39.72	16.84	11.80	27.95	76.63	24.53	11.25

**Table 5:** Results of the ferrous ions chelation capacity evaluation.

Compound	Chelation Capacity (%)								
	17.70 µg/mL	20.59 µg/mL	29.41 µg/mL	44.11 µg/mL	58.82 µg/mL	88.23 µg/mL	117.64 µg/mL	257.46 µg/mL	343.28 µg/mL
**5a**	-	-	-	-	18.44	34.12	45.93	71.76	86.30
**5b**	-	-	-	-	-	-	-	19.02	35.16
**5c**	-	-	-	-	-	-	20.27	65.75	89.24
**5d**	-	-	-	-	-	-	58.18	71.56	80.50
**6a**	-	-	-	-	-	-	-	-	-
**6b**	-	-	-	-	-	-	-	-	-
**6c**	-	-	-	-	-	-	-	-	-
**6d**	-	-	-	-	-	-	-	-	-
**EDTA-Na_2_**	1.32	20.59	42.89	95.10	+	+	+	+	+

- represents less than 5%; + represents more than 95%.

**Table 6:** Results of the cupric ions chelation capacity evaluation.

Compound	Chelation Capacity (%)		
	3.36 µg/mL	8.40 µg/mL	16.80 µg/mL
**5a**	20.33	30.76	40.29
**5b**	11.17	25.27	40.20
**5c**	13.06	23.23	32.60
**5d**	14.19	28.04	41.42
**6a**	10.96	20.64	31.79
**6b**	6.77	17.17	30.49
**6c**	7.47	16.75	26.72
**6d**	5.98	11.84	20.42
**EDTA-Na_2_**	10.39	22.68	44.51

**Table 7:** The energy of the frontier orbitals and the BDE for the H_1_-H_5_ sites in **5a**–**d** and **6a**–**d** compounds, according to the numeration presented in Figure 1.

Compound	Frontier Orbitals (eV)			X-H BDE (kcal/mol)				
	HOMO	LUMO	gap	H_1_	H_2_	H_3_	H_4_	H_5_
**5a**	−5.52	−1.62	3.90	78.08	72.00	78.27	88.90	101.09
**5b**	−5.53	−1.64	3.89	77.95	71.99	78.28	86.01	100.98
**5c**	−5.53	−1.60	3.93	77.98	71.97	78.26	89.91	106.64
**5d**	−5.51	−1.58	3.93	78.03	71.98	78.28	89.63	101.03
**6a**	−5.42	−1.58	3.84	84.54	N/A	79.45	83.21	N/A
**6b**	−5.39	−1.64	3.75	84.61	N/A	79.49	83.39	N/A
**6c**	−5.40	−1.57	3.83	84.64	N/A	79.45	83.19	N/A
**6d**	−5.39	−1.55	3.84	84.44	N/A	79.21	83.12	N/A

**Table 8:** The conformation with the lowest energy of the **5a**–**d** and **6a**–**d** compounds with the depiction of the resulting hydrogen bonds.

Compound	Conformation	Compound	Conformation
**5a**	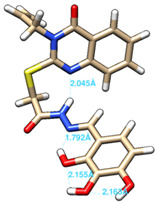	**6a**	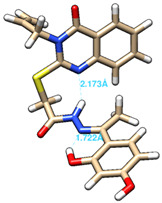
**5b**	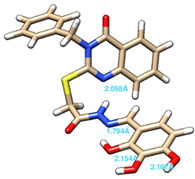	**6b**	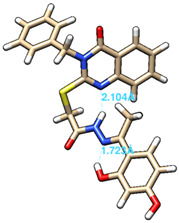
**5c**	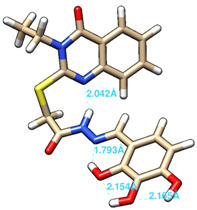	**6c**	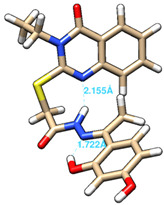
**5d**	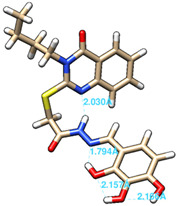	**6d**	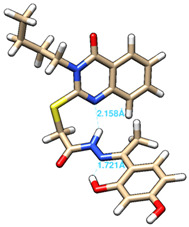

**Table 9:** Calculated IC_50_ values (µg/mL) after exposure of human lung adenocarcinoma (A549), human prostate carcinoma (LNCaP), and human normal foreskin fibroblasts (BJ) to the synthesized compounds (**5a**–**d** and **6a**–**d**), for 24 h.

Cell Line	IC_50_ (µg/mL)								
	Compounds								Reference
	5a	5b	5c	5d	6a	6b	6c	6d	Doxorubicin
**A549**	30.91	69.49	49.09	42.14	35.68	42.19	>100	32.42	0.54
**LNCaP**	45.64	69.97	35.92	56.80	43.37	30.40	>100	24.27	0.74
**BJ**	>100	>100	>100	>100	>100	>100	>100	>100	>7.5

**Table 10:** Molecular properties of the compounds **5a**–**d** and **6a**–**d** with strong influence on their pharmacokinetics.

Compound	MW	Rotatable Bonds	HBA	HBD	TPSA (Å^2^)	MLogP	Solubility (µg/mL)	Lipinski Violations
**5a**	426.45	8	7	4	162.34	1.29	61.10	0
**5b**	476.50	8	7	4	162.34	2.02	7.91	0
**5c**	414.44	7	7	4	162.34	0.72	86.80	0
**5d**	442.49	9	7	4	162.34	1.17	25.00	0
**6a**	424.47	8	6	3	142.11	2.02	27.00	0
**6b**	474.53	8	6	3	142.11	2.73	3.54	0
**6c**	412.46	7	6	3	142.11	1.46	38.90	0
**6d**	440.52	9	6	3	142.11	1.90	11.00	0

MW: molecular weight, HBA: hydrogen bond acceptors, HBD: hydrogen bond donors, TPSA: topological polar surface area.

## Data Availability

Not applicable.

## Supplementary Materials

The following supporting information can be downloaded at: https://www.mdpi.com/article/10.3390/pharmaceutics15010136/s1. Figures S1–S8: The IR spectrums for the compounds **5a**–**d** and **6a**–**d**; Figures S9–S16: The MS spectrums for the compound **5a**–**d** and **6a**–**d**; Figures S17–S24: The ^1^H-NMR spectrum for the compound **5a**–**d** and **6a**–**d**; Figures S25–S32: The ^13^C-NMR spectrum for the compound **5a**–**d** and **6a**–**d**; Table S1: The depiction of HOMO and LUMO for the compounds **5a**–**d** and **6a**–**d**; Table S2: The depiction of the spin density maps of the phenol group radicalization for the compounds **5a**–**d** and **6a**–**d**.
