# Supplementary material for: Antioxidant and Cytotoxic Activity of New Polyphenolic Derivatives of Quinazolin-4(3H)-one: Synthesis and In Vitro Activities Evaluation
Source: Pharmaceutics. 2022 Dec 30;15(1):136. doi: 10.3390/pharmaceutics15010136 (PMC9867241; doi:10.3390/pharmaceutics15010136)
Supplement: Supplementary file 1 [file pharmaceutics-15-00136-s001.zip › pharmaceutics-2116218-supplementary.pdf]

pharmaceutics

MDPI

Article

Supplementary Material: Antioxidant and Cytotoxic Activity of New Polyphenolic Derivatives of Quinazolin-4(3H)-one: Synthesis and In Vitro Activities Evaluation

Raluca Pele, Gabriel Marc, Ioana Ionuț, Cristina Nastasă, Ionel Fizeșan, Adrian Pîrnău, Laurian Vlase, Mariana Palage, Smaranda Oniga and Ovidiu Oniga

1. Figures

1.1. The IR spectra

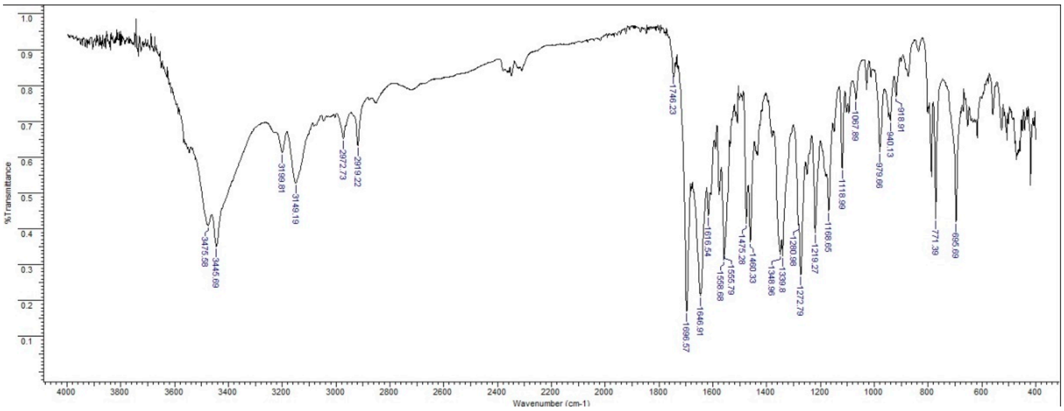

Figure S1. The IR spectrum for the compound **5a.**

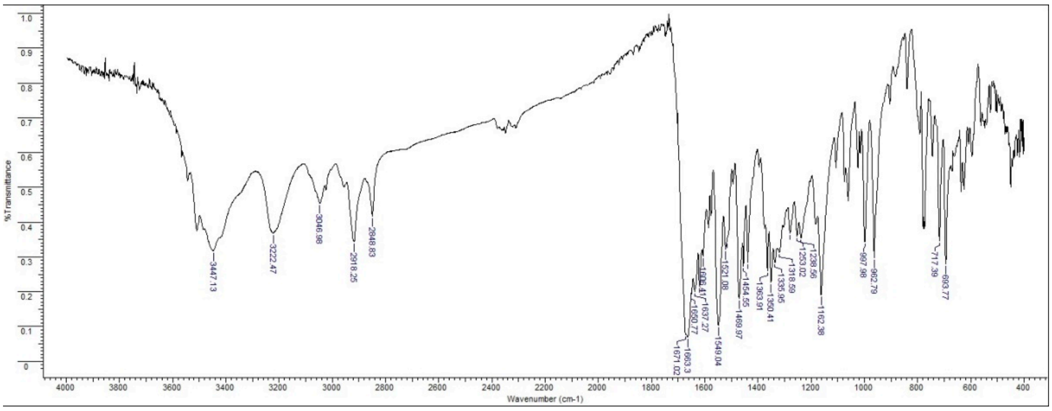

Figure S2. The IR spectrum for the compound **5b.**

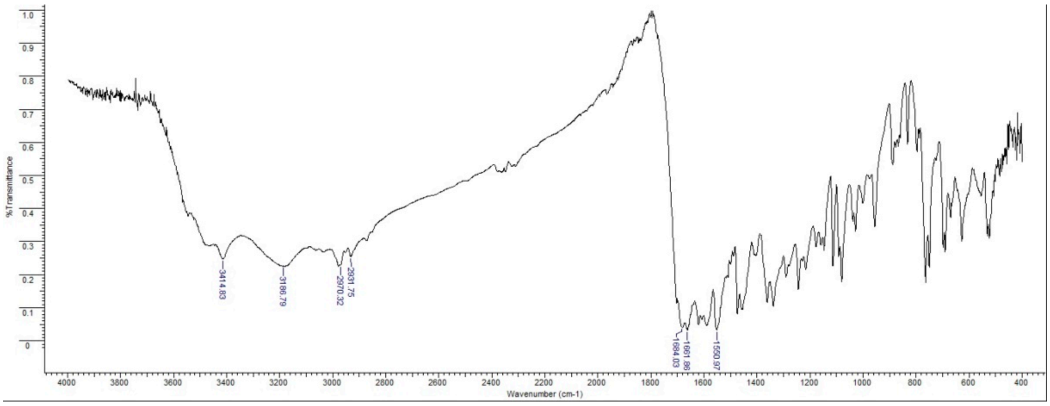

Figure S3. The IR spectrum for the compound **5c.**

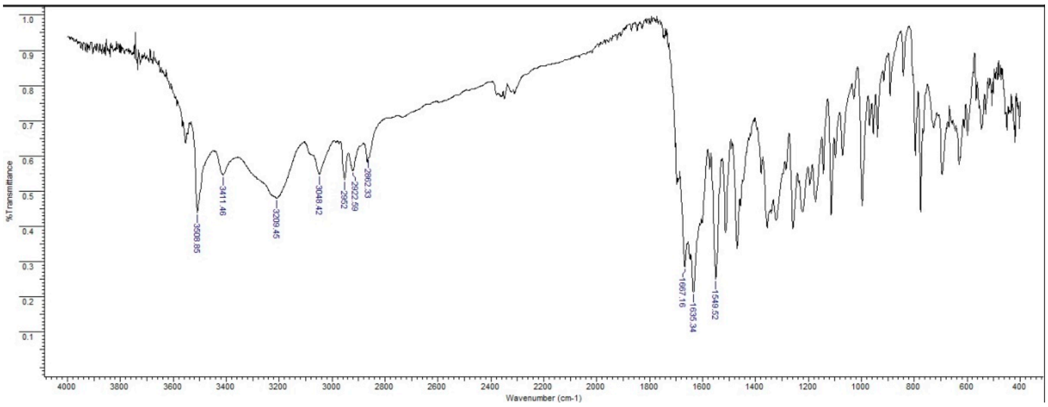

Figure S4. The IR spectrum for the compound **5d.**

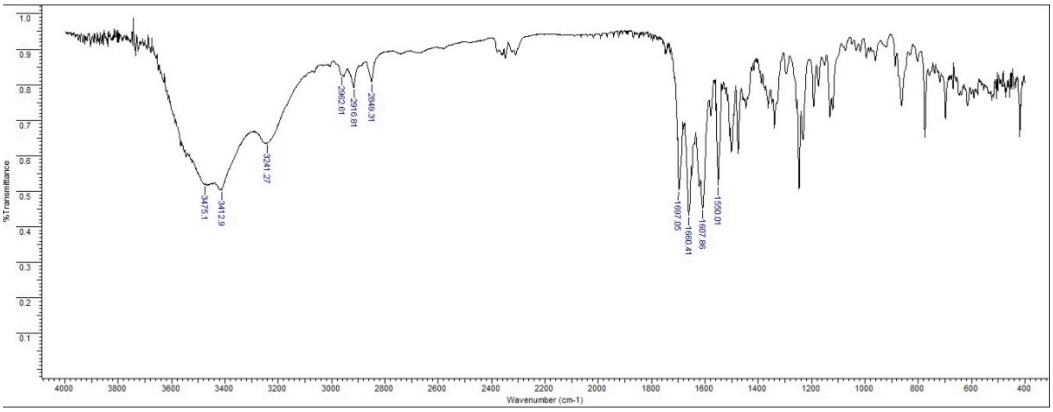

Figure S5. The IR spectrum for the compound **6a.**

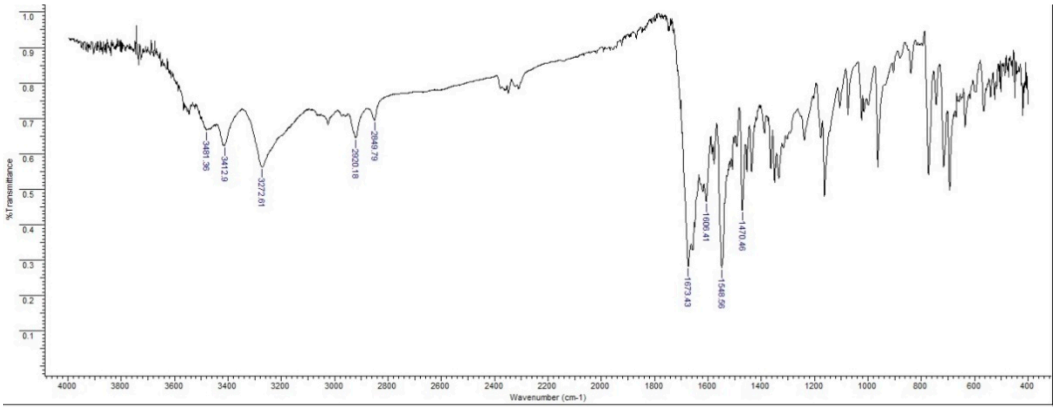

Figure S6. The IR spectrum for the compound **6b.**

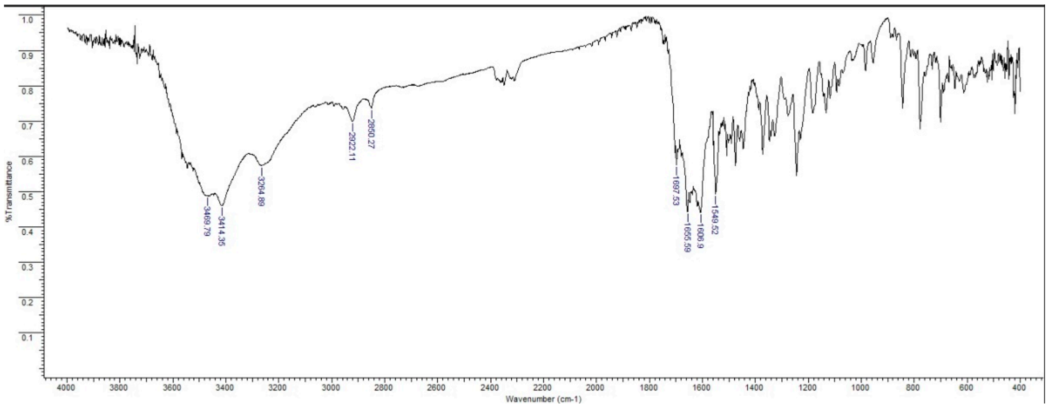

Figure S7. The IR spectrum for the compound **6c.**

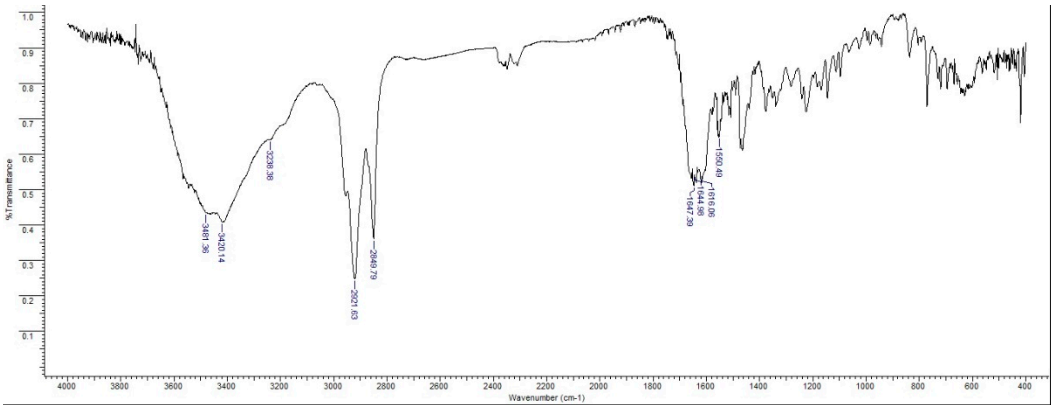

Figure S8. The IR spectrum for the compound **6d.**

1.2. The MS spectra

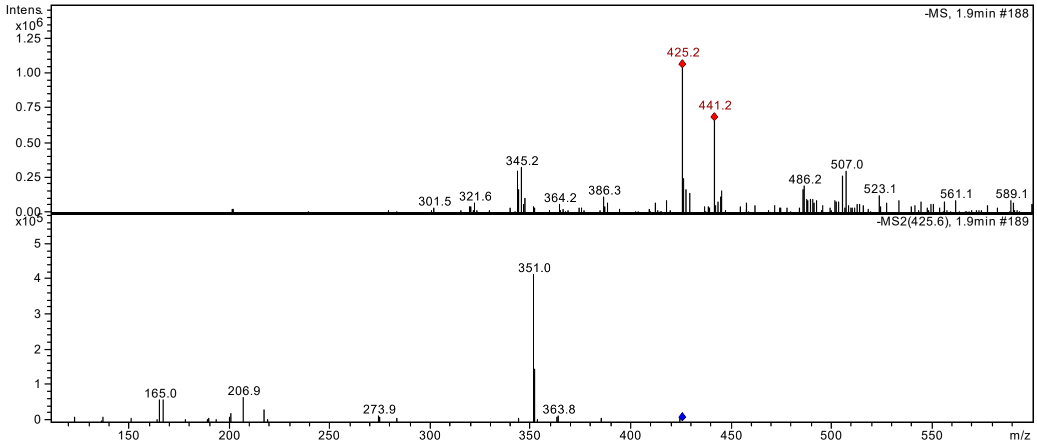

Figure S9. The MS spectrum for the compound **5a.**

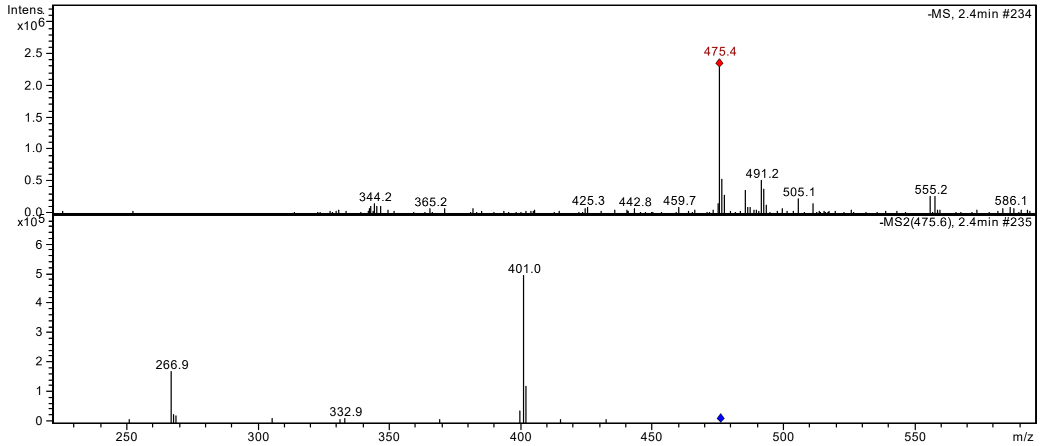

Figure S10. The MS spectrum for the compound **5b.**

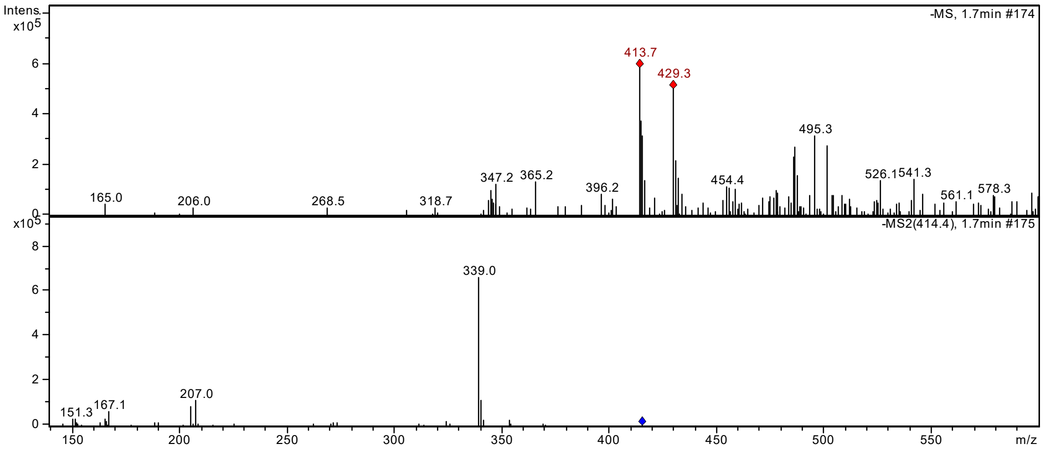

Figure S11. The MS spectrum for the compound **5c.**

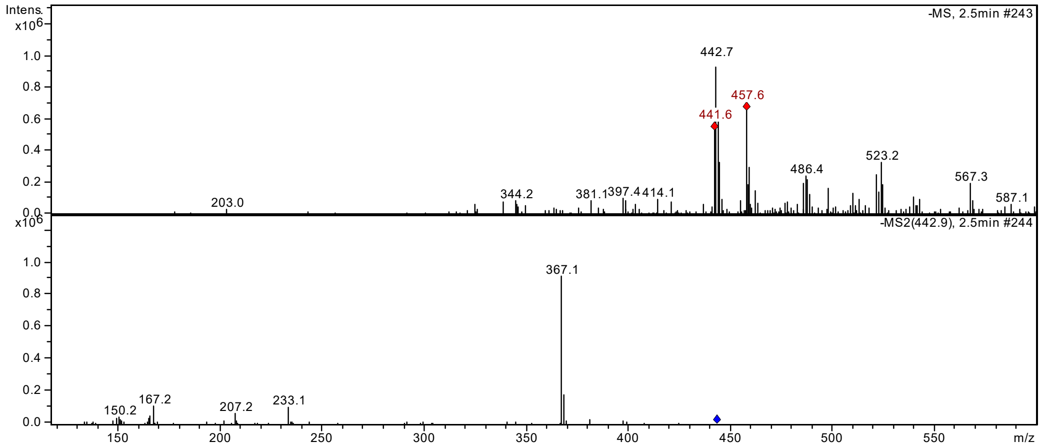

Figure S12. The MS spectrum for the compound **5d.**

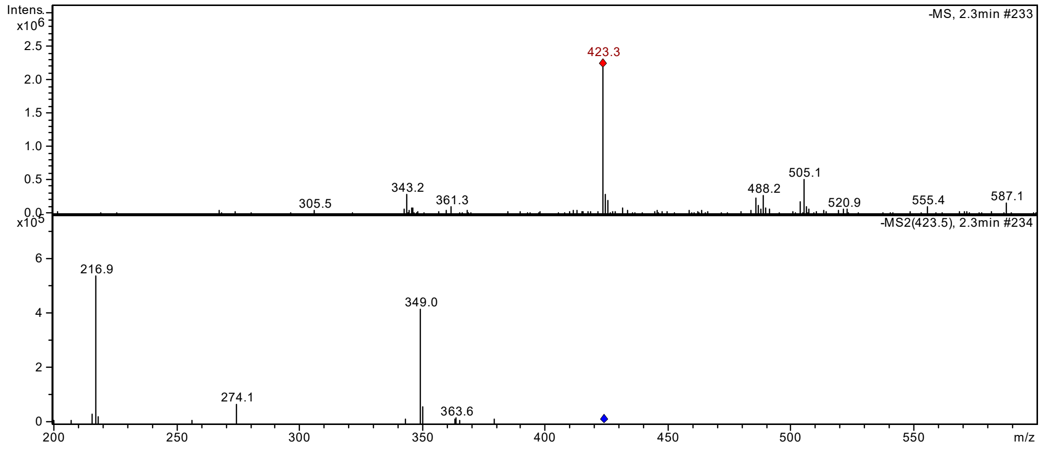

Figure S13. The MS spectrum for the compound **6a.**

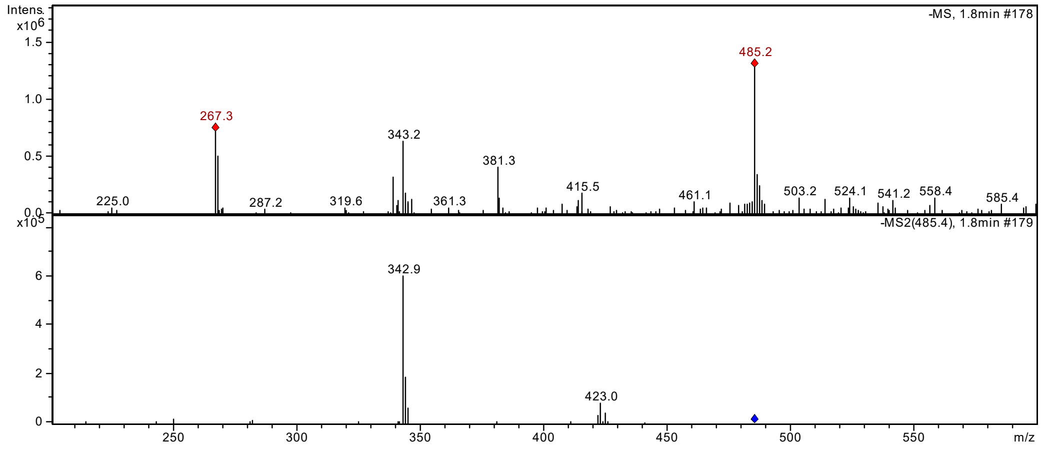

Figure S14. The MS spectrum for the compound **6b.**

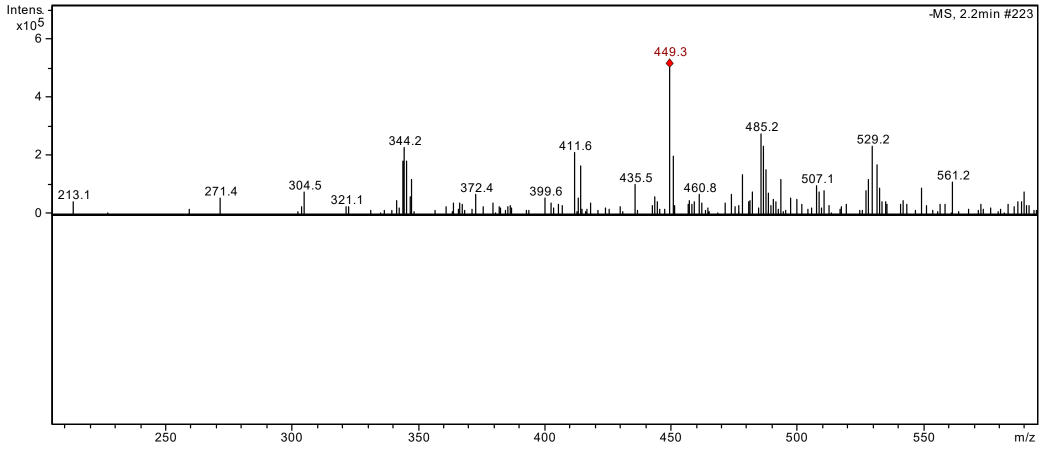

Figure S15. The MS spectrum for the compound **6c.**

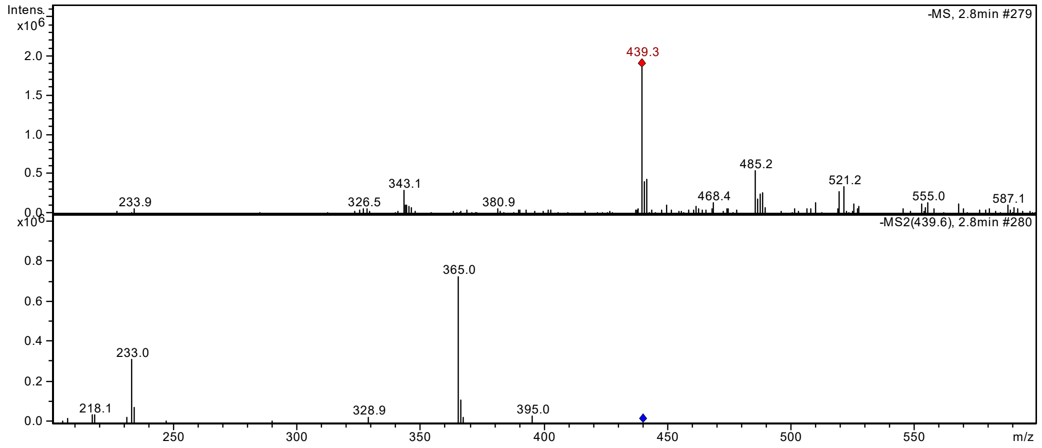

Figure S16. The MS spectrum for the compound **6d.**

1.1. *The ^{1}H-NMR spectrum*

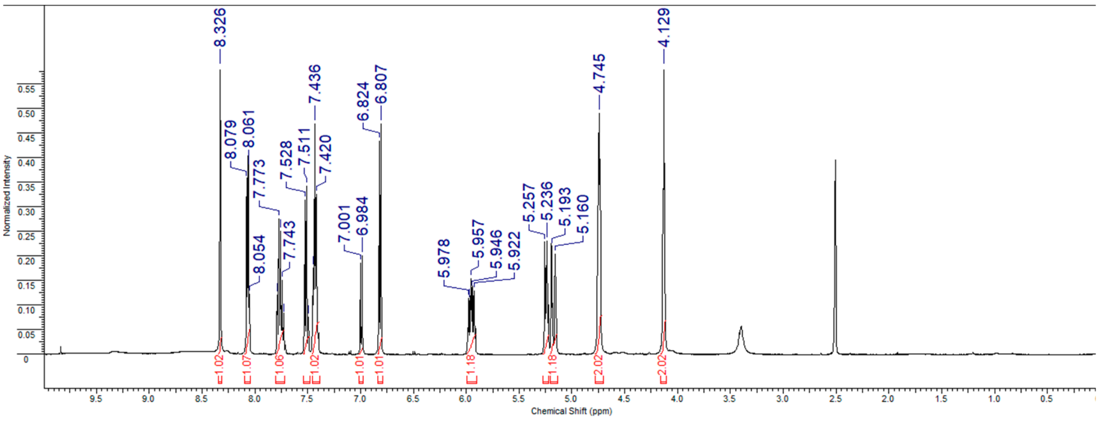

Figure S17. The ^{1}H-NMR spectrum for the compound **5a.**The integral label for the triplet at 7.511 ppm is 1.04 and for the multiplet at 5.239 ppm is 1.04.

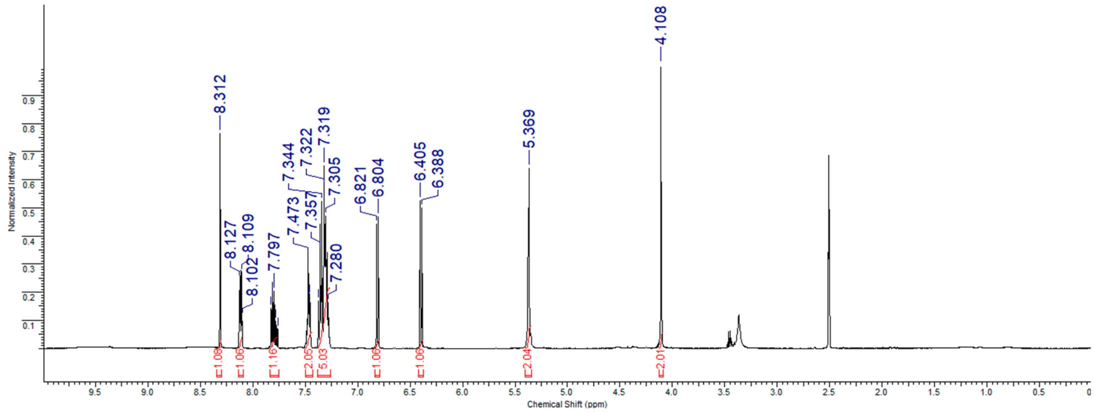

Figure S18. The ^{1}H-NMR spectrum for the compound **5b.**

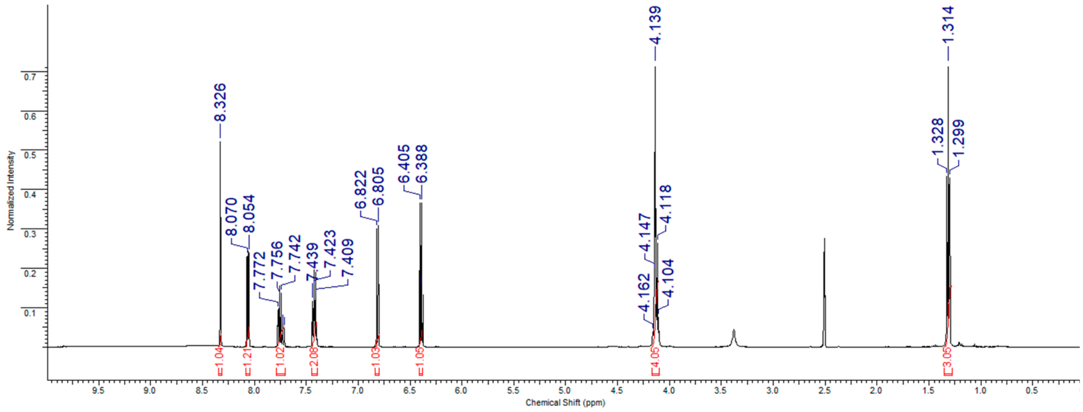

Figure S19. The ^{1}H-NMR spectrum for the compound **5c.**

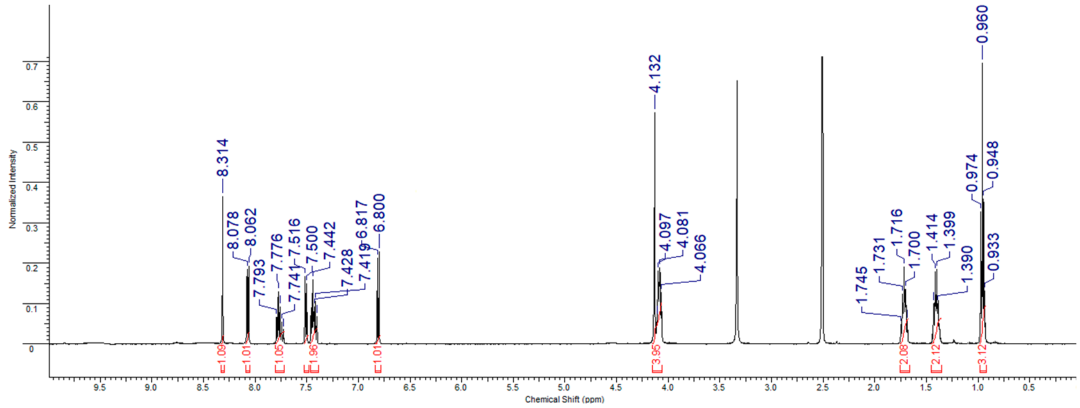

Figure S20. The ^{1}H-NMR spectrum for the compound **5d.** The integral label for the doublet at 7.508 ppm is 1.09.

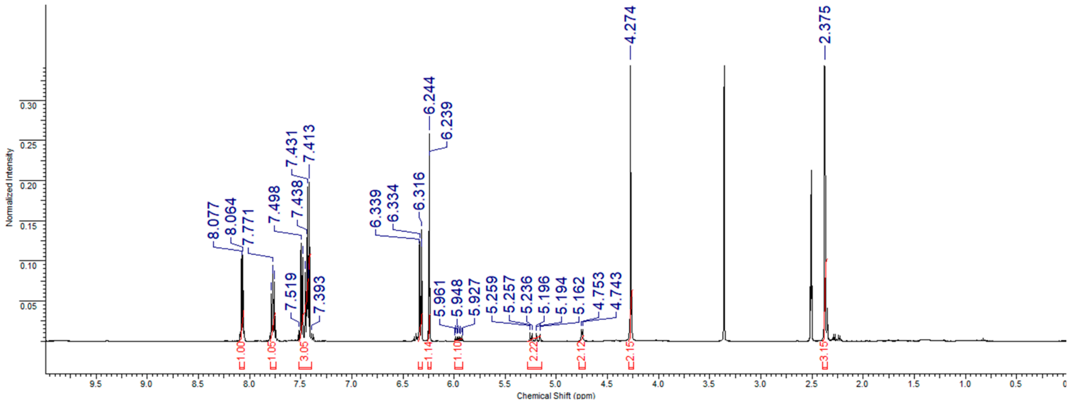

Figure S21. The ^{1}H-NMR spectrum for the compound **6a.** The integral label for the doublet of doublets at 6.327 ppm is 1.19.

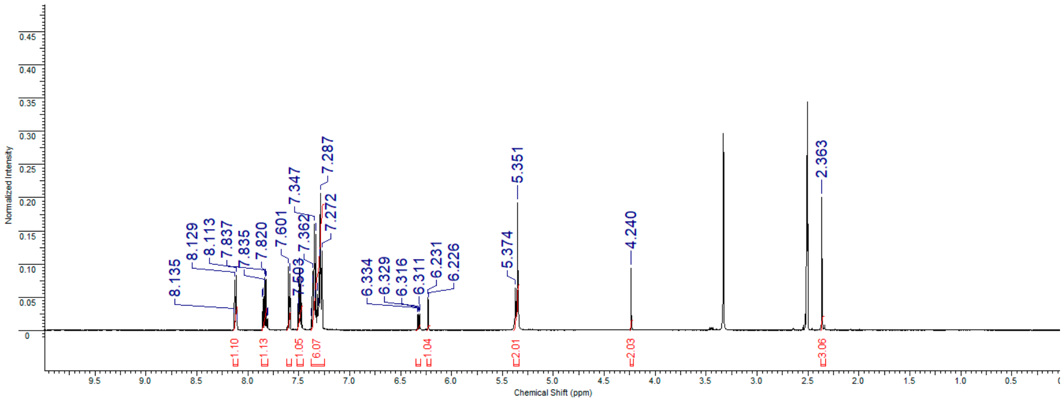

Figure S22. The ^{1}H-NMR spectrum for the compound **6b.** The integral label for the doublet at 7.593 ppm is 0.90 and for the doublet of doublets at 6.322 ppm is 1.02.

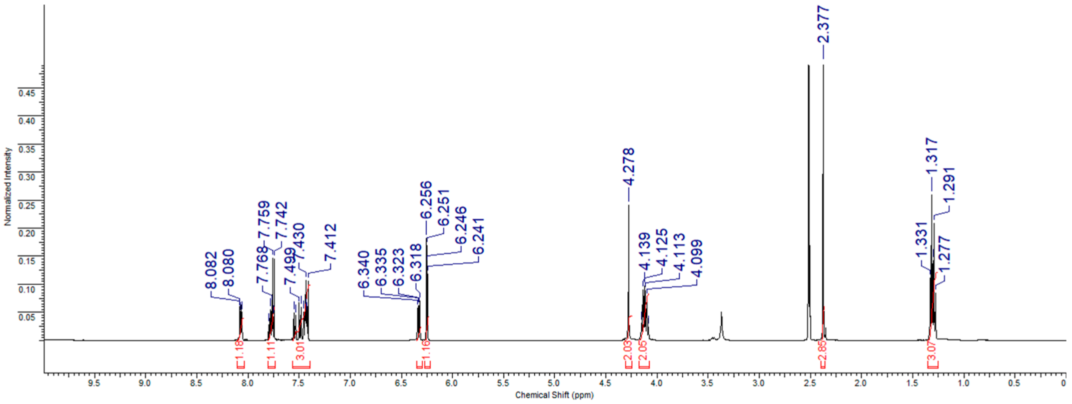

Figure S23. The ^{1}H-NMR spectrum for the compound **6c.** The integral label for the doublet of doublets at 6.328 ppm is 0.90.

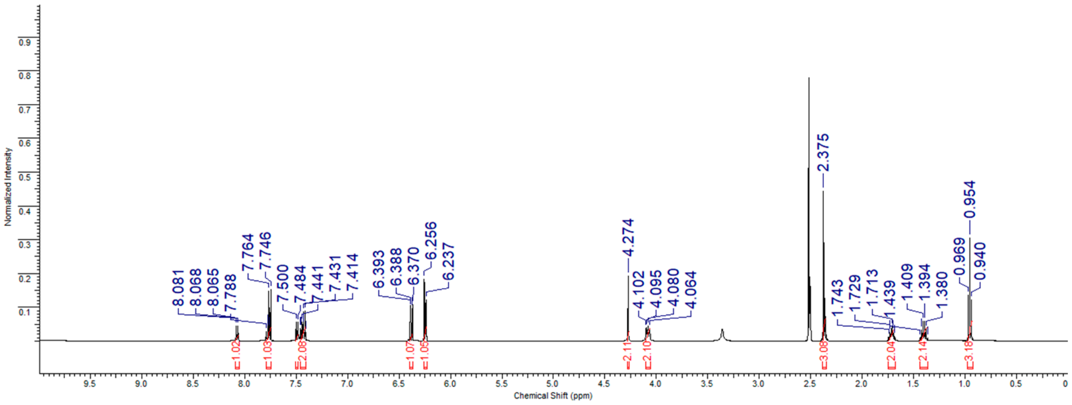

Figure S24. The ^{1}H-NMR spectrum for the compound **6d.** The integral label for the doublet at 7.492 ppm is 1.13.

1.2. The ^{13}C-NMR spectra

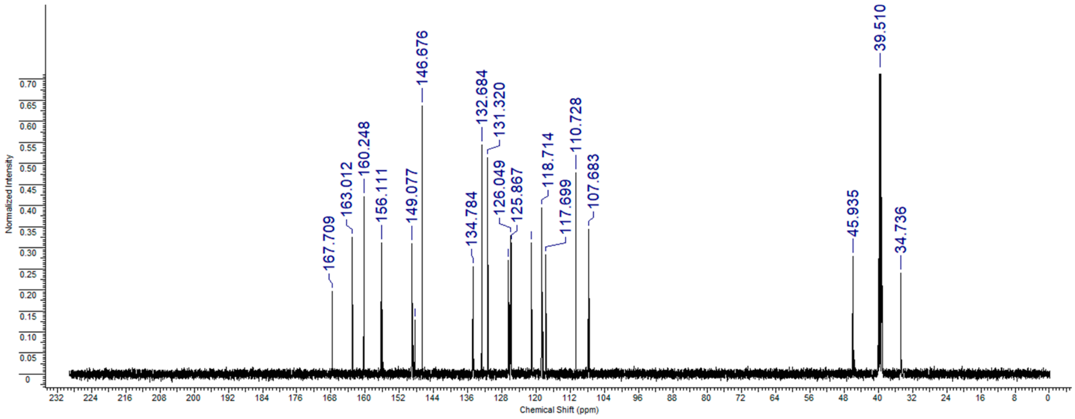

Figure S25. The ^{13}C-NMR spectrum for the compound **5a.**

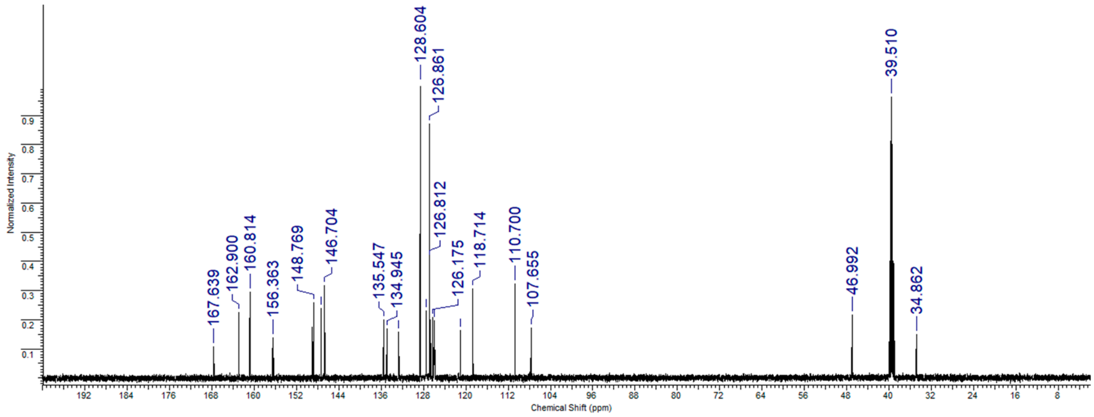

Figure S26. The ^{13}C-NMR spectrum for the compound **5b.**

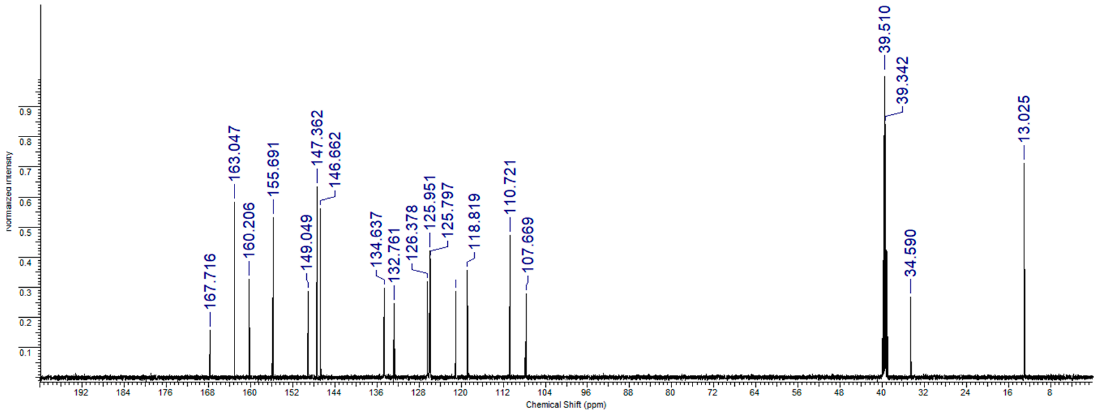

Figure S27. The ^{13}C-NMR spectrum for the compound **5c.**

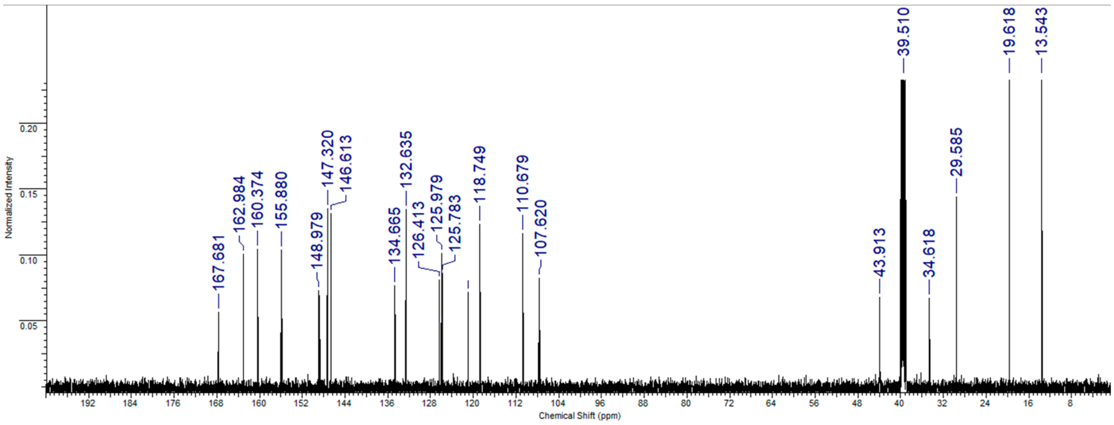

Figure S28. The ^{13}C-NMR spectrum for the compound **5d.**

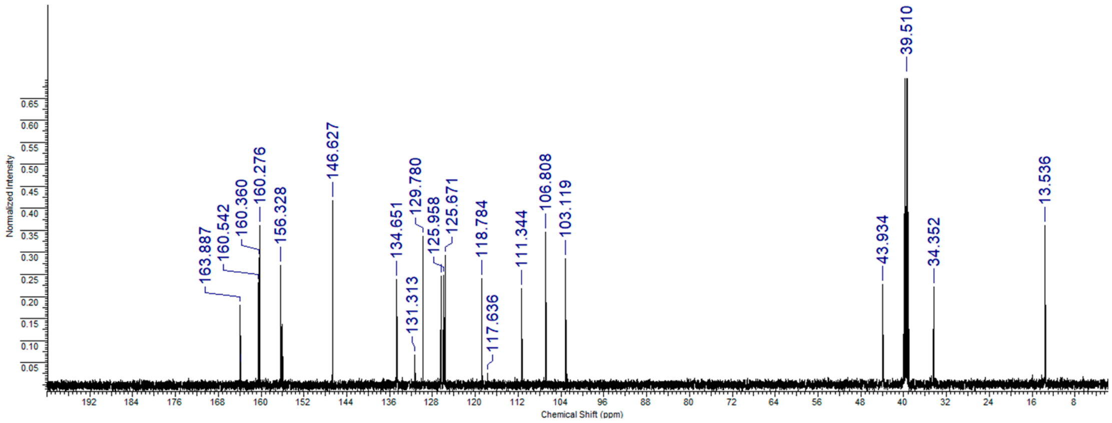

Figure S29. The ^{13}C-NMR spectrum for the compound **6a.**

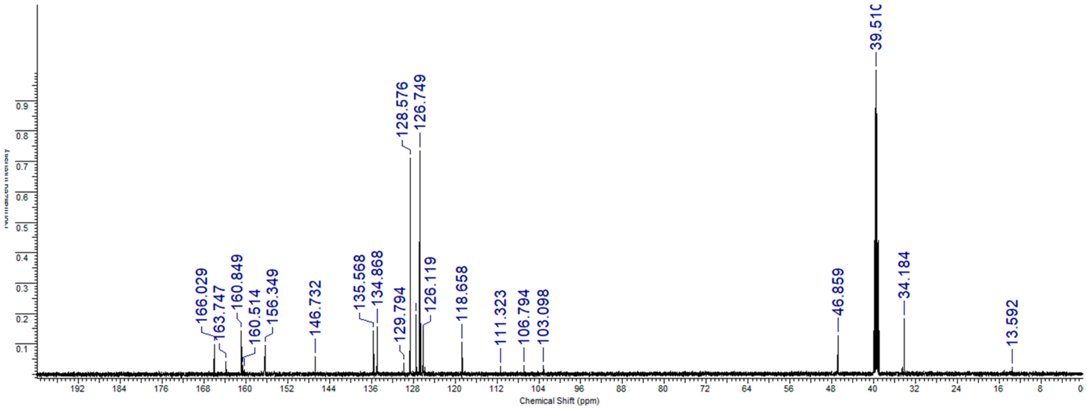

Figure S30. The ^{13}C-NMR spectrum for the compound **6b.**

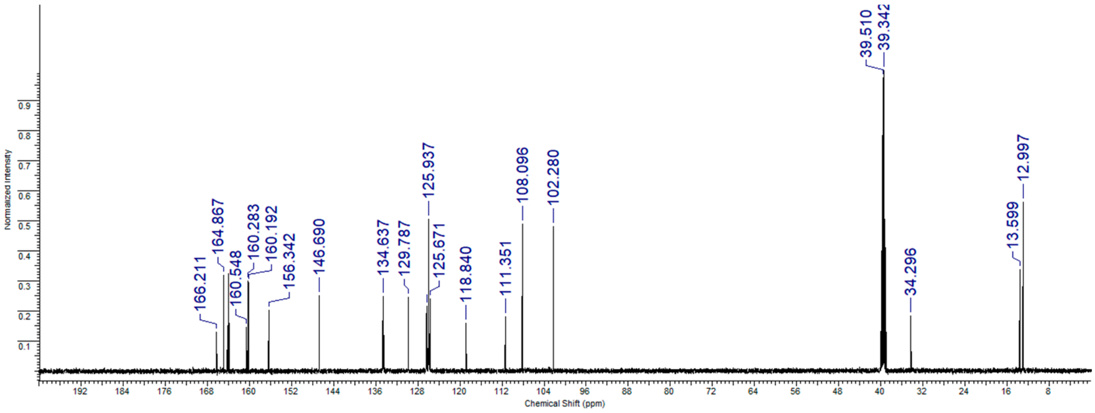

Figure S31. The ^{13}C-NMR spectrum for the compound **6c.**

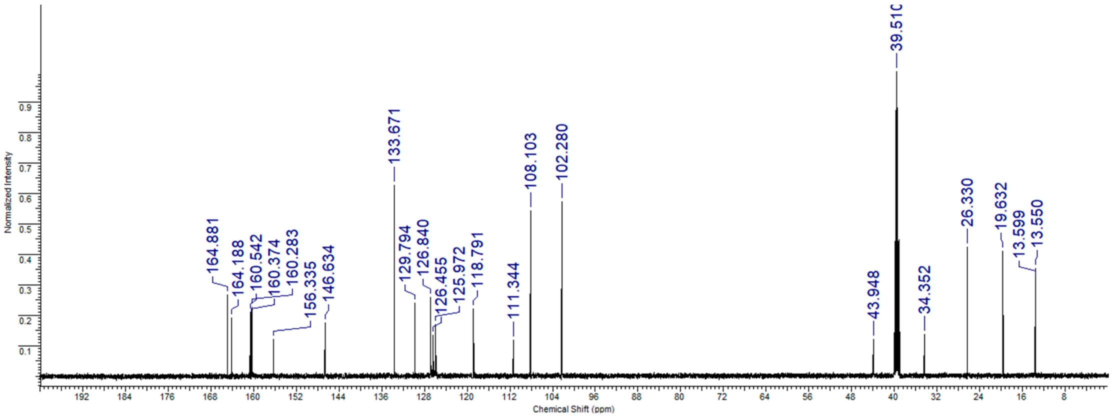

Figure S32. The ^{13}C-NMR spectrum for the compound **6d.**

2. Tables

*2.1. The depiction of HOMO and LUMO and the depiction of the spin density maps of the phenol group radicalization for the compounds **5a-d** and **6a-d***

Table S1. The depiction of HOMO and LUMO for the compounds **5a-d** and **6a-d**

Compound	HOMO	LUMO

Compound	HOMO	LUMO
**5a**	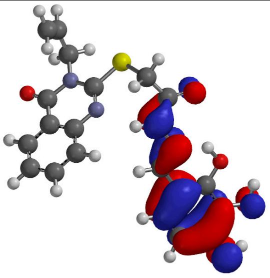	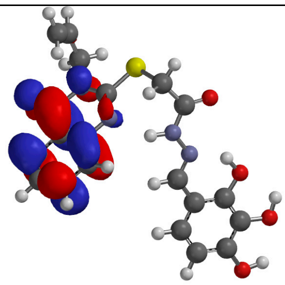
**5b**	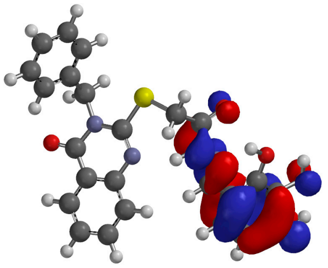	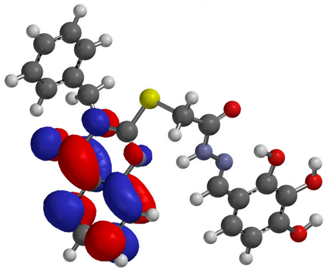
**5c**	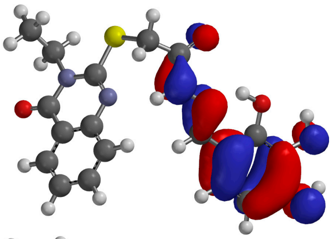	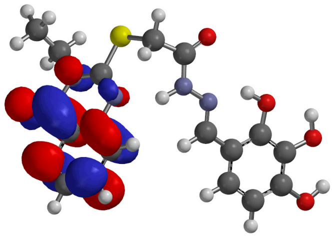
**5d**	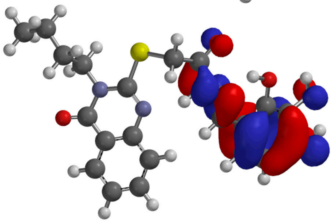	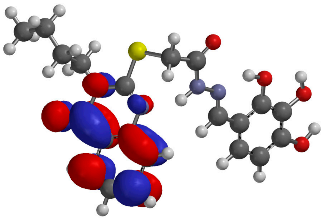
**6a**	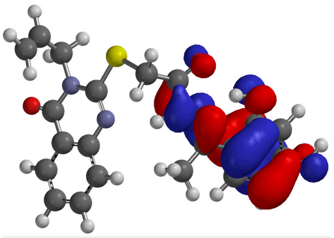	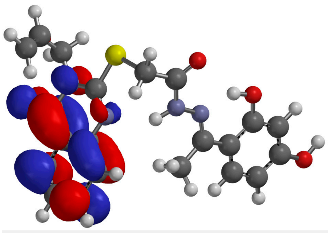
**6b**	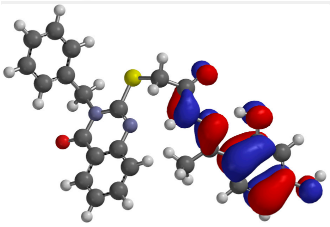	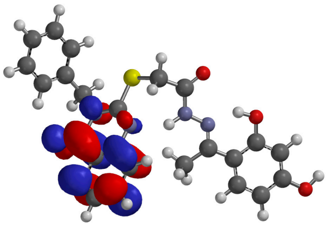
**6c**	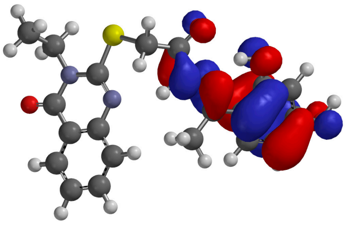	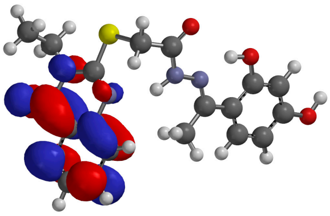

Compound	HOMO	LUMO
**6d**	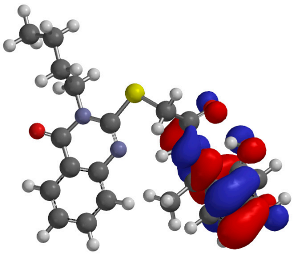	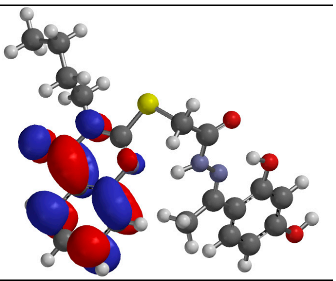

Table S2. The depiction of spin density maps for the phenol group radicalization for the compounds **5a-d** and **6a-d**

Compound	Spin density map depending on the phenolic radical		
	ortho (H1)	meta (H2)	para (H3)
**5a**	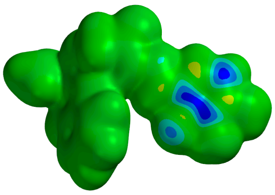	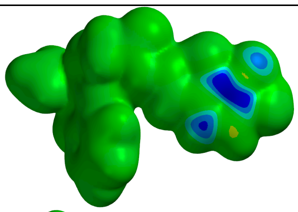	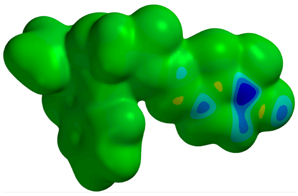
**5b**	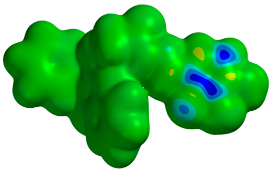	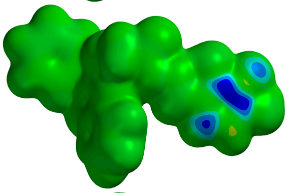	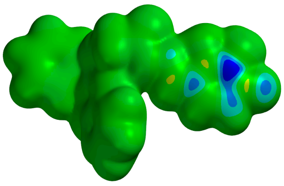
**5c**	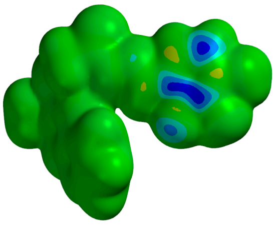	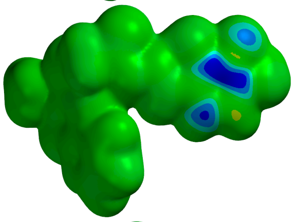	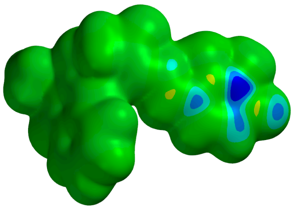
**5d**	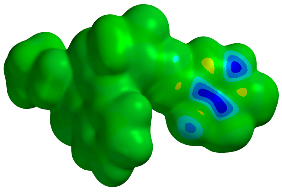	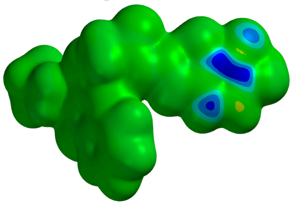	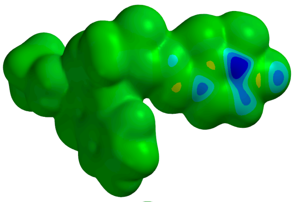
**6a**	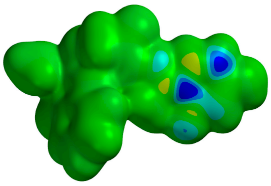	N/A	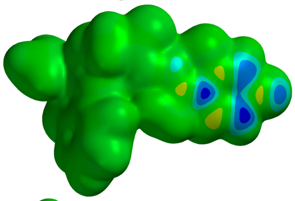
**6b**	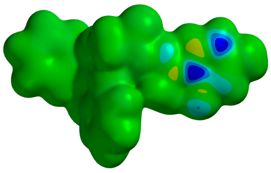	N/A	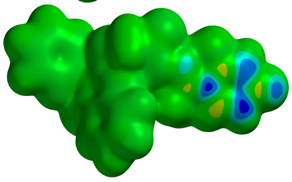

Compound	Spin density map depending on the phenolic radical		
	ortho (H1)	meta (H2)	para (H3)
6c	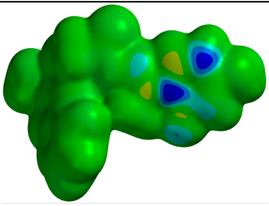	N/A	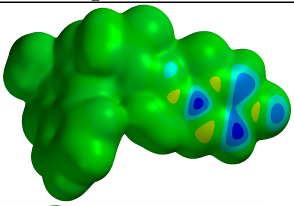
6d	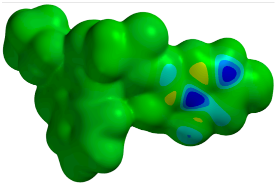	N/A	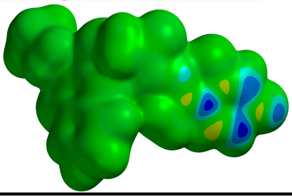
